# ERP Correlates of Altered Orthographic-Phonological Processing in Dyslexia

**DOI:** 10.3389/fpsyg.2021.723404

**Published:** 2021-10-13

**Authors:** Vera Varga, Dénes Tóth, Kathleen Kay Amora, Dávid Czikora, Valéria Csépe

**Affiliations:** ^1^Brain Imaging Centre, Research Centre for Natural Sciences, Budapest, Hungary; ^2^Department of Cognitive Science, Budapest University of Technology and Economics, Budapest, Hungary; ^3^Multilingualism Doctoral School, Faculty of Modern Philology and Social Sciences, University of Pannonia, Veszprém, Hungary; ^4^Institute for Hungarian and Applied Linguistics, Pannon University, Veszprém, Hungary

**Keywords:** dyslexia, N170 effect, position coding, ERP, audiovisual processing, print sensitivity

## Abstract

Automatic visual word recognition requires not only well-established phonological and orthographic representations but also efficient audio-visual integration of these representations. One possibility is that in developmental dyslexia, inefficient orthographic processing might underlie poor reading. Alternatively, reading deficit could be due to inefficient phonological processing or inefficient integration of orthographic and phonological information. In this event-related potential study, participants with dyslexia (*N* = 25) and control readers (*N* = 27) were presented with pairs of words and pseudowords in an implicit same-different task. The reference-target pairs could be identical, or different in the identity or the position of the letters. To test the orthographic-phonological processing, target stimuli were presented in visual-only and audiovisual conditions. Participants with and without dyslexia processed the reference stimuli similarly; however, group differences emerged in the processing of target stimuli, especially in the audiovisual condition where control readers showed greater N1 responses for words than for pseudowords, but readers with dyslexia did not show such difference. Moreover, after 300 ms lexicality effect exhibited a more focused frontal topographic distribution in readers with dyslexia. Our results suggest that in developmental dyslexia, phonological processing and audiovisual processing deficits are more pronounced than orthographic processing deficits.

## Introduction

Automatic visual word recognition requires not only well-established phonological and orthographic representations but also efficient audio-visual integration of these representations. Most children acquire these skills without any problems; however, around 5–10% of school-aged children fail to develop age-appropriate reading and spelling skills (Schulte-Körne, 2010; Galuschka and Schulte-Körne, 2016; Barbiero et al., 2019). Developmental dyslexia is characterized by a specific impairment in reading despite normal IQ, lack of any specific sensory impairment and adequate education (American Psychiatric Association, 2013). According to the phonological processing deficit hypothesis (Vellutino et al., 2004), the core problem of dyslexia is poor phonological processing which can manifest as impaired grapheme-phoneme mapping (Blomert, 2011). Skilled adult readers typically exhibit automatic grapheme-phoneme mapping wherein presentation of one code activates the other and vice versa (Harm et al., 2004); however, in readers with dyslexia speech-sound associations may never reach automatization (Vellutino et al., 2004). Thus, phonological dyslexia is characterized by impaired pseudoword reading with relatively normal word reading.

Characteristics of reading impairment may vary in dyslexia depending on the orthographic transparency of languages. In opaque orthographies reading accuracy seems to be impaired in dyslexia (English: Landerl et al., 1997; Ziegler et al., 2003), whereas in semi-transparent (German: Landerl et al., 1997; Wimmer and Schurz, 2010; Dutch: Verhoeven and Keuning, 2018) and in transparent orthographies (Spanish: Serrano and Defior, 2008; Italian: Tressoldi et al., 2001; Hungarian: Csépe et al., 2003; Landerl et al., 2013; Mohai, 2014; Finnish: Eklund et al., 2015) mainly slow reading times of pseudowords as well as spelling difficulties (Spanish: Afonso et al., 2015; German: Galuschka and Schulte-Körne, 2016) characterize the impairment. Slow pseudoword reading times may be the result of impaired grapheme-phoneme integration, whereas spelling deficits might suggest an orthographic impairment, as well. To expound this further, we will first review what is known about orthographic processing in dyslexia. Then, we will summarize the orthographic-phonological integration deficit related to dyslexia.

Extensive experience with orthographic stimuli results in highly specialized perception for print. Visually presented orthographic stimuli (e.g., words, pseudowords, consonant strings) evoke a negative peak in adults around 150–200 ms after stimulus onset over occipito-temporal brain regions. This electrophysiological component is called the N1 or N170 response (Bentin et al., 1999) and is considered to be the functional correlate of visual expertise for print. It seems that two levels of print sensitivity exists: (1) a fast, coarse-grade print sensitivity for print indexed by different processing of letter strings compared to control visual stimuli such as symbol strings or false fonts (early N1) and (2) a fine-grade print sensitivity for orthographically familiar letter sequences such as words compared to unfamiliar sequences such as pseudowords or non-words (late N1, see Eberhard-Moscicka et al., 2016).

Letter strings exhibit enhanced N1 response compared to symbol strings or false fonts (Bentin et al., 1999; Maurer et al., 2005a,b, 2010). This coarse-grade sensitivity emerges during reading acquisition. Although this is absent in kindergarten children (Maurer et al., 2005b), it emerges after one year of reading instruction (Eberhard-Moscicka et al., 2015; Varga et al., 2020) and follows an inverted U shape pattern which peaks during reading acquisition and then declines over instruction (Fraga-González et al., 2021). The N1 for print is more pronounced over the left posterior-occipital regions (Maurer et al., 2005a; Yoncheva et al., 2010), and this left lateralization is enhanced with reading experience. According to the phonological mapping hypothesis, the left lateralization is driven by automatized grapheme-phoneme mapping (Maurer et al., 2007). Typically, children show a bilateral effect for letter strings (Maurer et al., 2006; Kast et al., 2010); however, recent studies found that left lateralization can be found as early as one year (Varga et al., 2020; van de Walle de Ghelcke et al., 2021) or even half a year of reading instruction (Pleisch et al., 2019; altough lateralization is less clear for single letters, see Fraga-González et al., 2021).

As the above results indicate that reading acquisition and reading practice heavily influence coarse-grained sensitivity for print, but the presence of this print sensitivity in individuals with dyslexia is widely debated. A number of studies found evidence for attenuated N1 for print in children (Maurer et al., 2007; Araújo et al., 2012) or adults with dyslexia (Helenius et al., 1999; Mahé et al., 2012, 2013), but some studies failed to find any difference in print sensitivity between children (Hasko et al., 2012) or adults with and without dyslexia (Araújo et al., 2015). Studies reporting N1 impairments in dyslexia (Helenius et al., 1999; Mahé et al., 2012, 2013) usually included participants with more severe reading deficits compared to the control group suggesting that the degree of reading and spelling impairments can influence orthographic deficits (Mahé et al., 2012).

The fine-grade sensitivity or lexical sensitivity of the N1 is less robust and more task-dependent than coarse-grade print sensitivity. Some studies found greater N1 for pseudowords compared to words (Sereno et al., 1998; Hauk and Pulvermüller, 2004; Hauk et al., 2006; Dujardin et al., 2011; Araújo et al., 2015), while some others found greater N1 for words compared to pseudowords (Maurer et al., 2006; Kast et al., 2010; Eberhard-Moscicka et al., 2016; Faísca et al., 2019) suggesting that top-down linguistic information modulates early orthographic processing. Contrary to these results, other studies failed to find differences between the processing of word and pseudoword stimuli (Maurer et al., 2005b; Araújo et al., 2012; Hasko et al., 2013; Eberhard-Moscicka et al., 2015, 2016) suggesting that the N1 component arises at the prelexical stage of orthographic processing and is sensitive to orthographic but not to lexical constraints. The inconsistency of results probably arises due to developmental effects (adolescent: Araújo et al., 2012; grade 2: Maurer et al., 2006), differences between orthographic transparency of the language investigated (French: Bentin et al., 1999; English: Maurer et al., 2005a; Hauk et al., 2006; German: Maurer et al., 2005b), and task demands (Maurer and McCandliss, 2007; Faísca et al., 2019). Maurer and McCandliss (2007) proposed that word vs. pseudoword differences emerge when grapheme-phoneme mapping is not automatic. This argument is supported by results that show N1 fine tuning mainly in implicit reading tasks where grapheme-phoneme mapping is not required. In addition, even in these tasks, fine tuning is mostly present for readers of deep orthographies like English (Maurer et al., 2005a) and novice readers (Maurer et al., 2006).

Previous results on readers with dyslexia are even more ambiguous. For instance, 7 years old children with dyslexia showed decreased N1 amplitude to pseudowords but not to words compared to controls in one study (Wimmer et al., 2002), while adult with dyslexia showed similar lexicality effect as typical readers in another (larger N1 for pseudowords compared to words, Araújo et al., 2015). Furthermore, the results of Mahé et al. (2012) suggest that skilled adult readers show lexicality effect in the left hemisphere, while adults with dyslexia showed no lexicality effect. Finally, Kast et al. (2010) found that typically developing children showed enhanced N1 amplitude for words compared to pseudowords in a lexical decision task. In comparison, children with dyslexia showed the opposite pattern of results, pseudowords elicited greater N1 than words. This could signify the enhanced effort to decode unfamiliar orthographic strings (pseudowords) in dyslexia. What seems to be less ambiguous, however, is that the N1 effect is usually less left-lateralized in readers with dyslexia than in skilled readers (adults: Helenius et al., 1999; Mahé et al., 2012; children: Kast et al., 2010; Araújo et al., 2012, but see Fraga-González et al., 2014).

On the other hand, for skilled reading it is not sufficient to efficiently categorize visual stimuli. Expert readers automatically identify letters and encode their position in the words they read (Grainger, 2008). This is essential in order to successfully recognize words from among the visually similar candidates (so called orthographic neighbors). For instance, to correctly recognize the word “CALM” readers must identify each letter and inhibit (substituted letter) neighbors like “CALF” or “PALM.” In addition, readers also need to process letter positions to inhibit (transposed letter) neighbors like “CLAM.” The latter can be problematic even for skilled readers as they sometimes confuse transposed letter words (transposed-letter effect, see Grainger, 2008). In fact, Castles et al. (2007) found that sensitivity to letter identity and letter position changes as a function of reading development. While third graders tolerate both letter identity and position mismatch between letter strings, fifth graders are sensitive to letter identity changes but still insensitive to letter position changes. Similarly, Tóth and Csépe (2017) demonstrated that children through 2nd to 4th grade show improvement in sensitivity for letter identity but not for letter position encoding.

There are two components (N1 and N250) reported in the literature that seem to capture fine orthographic differences between word pairs. First, between 100 and 200 ms after stimulus onset, the N1 component is believed to reflect visual perceptual discrimination (Vogel and Luck, 2000). In this time window, the degree of visual overlap between the prime and target items modulates ERP responses (Grainger and Holcomb, 2009; Duñabeitia et al., 2012). Thus, differences in letter identity or letter order between word pairs might result in an increased N1 response as these differences decrease the visual overlap between the word pairs. Indeed, this seems to be the case. For example, Cao et al. (2015) showed greater N1 for different word pairs than for identical word pairs. Furthermore, Duñabeitia et al. (2012) found greater N1 for targets including letter substitution compared to targets including letter transpositions. This later result suggests that letter substitution is visually more salient than letter transposition.

Second, a component between 200 and 325 ms is also sensitive to orthographic overlap (Grainger and Holcomb, 2009). The N250 peaks at around 250 ms and its distribution is largest over midline and anterior left sites. Holcomb and Grainger (2006) found for instance that N250 is greater when prime-target pairs differ in one-letter (substitution) than when they completely overlap (identical). Moreover, Duñabeitia et al. (2012) demonstrated a larger N250 for substituted letter strings in a same-different task compared to transposed letter strings (see also Dunabeitia et al., 2009). Finally, Holcomb and Grainger (2006) presented primes to their participants that could be identical, different in one substituted letter or completely different from the target. While only differences at the global word-form level were detected (identical vs. completely different pairs) in the N1 time-window (125–175 ms), finer word-form differences (identical vs. substituted letter pairs) modulated the N250 (175–300 ms) and the N400 (400–550 ms) components, too. The authors concluded that the ERP correlate of letter processing is the N250 component.

Although a number of studies examined orthographic processing in skilled readers, much less experiments investigated these processes in reading disorders. In their study, Ogawa et al. (2016) found impaired orthographic processing in adults with dyslexia. While typically reading Japanese children showed the Stroop effect for real words and their transposed-letter pseudoword pairs, readers with dyslexia showed the Stroop effect for real words only which suggests orthographic processing deficits. In another experiment Reilhac et al. (2012) compared the performance of children with and without dyslexia on a same-different task. Responses were more accurate when two letters were substituted rather than transposed in both groups. This substitution advantage was found in controls regardless of the lexicality of the letter string and was somewhat larger for pseudowords than word, but the effect was only present for words in children with dyslexia. In sum, it seems that readers with dyslexia have deficits in letter identity and position processing (Reilhac et al., 2012; Ogawa et al., 2016), but to our knowledge, no previous studies examined the electrophysiological correlates of letter identity and letter position encoding in individuals with dyslexia.

Though it seems that visual sensitivity for print (Maurer et al., 2007; Kast et al., 2010; Araújo et al., 2012; Mahé et al., 2012) and fundamental orthographic processes like letter identity and letter position encoding (Reilhac et al., 2012; Ogawa et al., 2016) can be affected in dyslexia, phonological deficits are usually more severe (Blomert, 2011; Lété and Fayol, 2013). In addition, even orthographic processes are thought to be tuned by phonology (Maurer and McCandliss, 2007; Meade, 2020); therefore, comparing deficits in orthographic processing and deficits in the integration of orthographic and phonological information is crucial.

In fact, numerous studies point to an audiovisual (AV) integration deficit in dyslexia (Froyen et al., 2011; Mingjin et al., 2012; Mittag et al., 2013; Hasko et al., 2014; Kronschnabel et al., 2014; Žarić et al., 2015; Wang et al., 2020; for a review see: Blomert, 2011). For instance, Froyen et al. (2011) reported that 11 year old children with dyslexia do not exhibit automatic integration of letters and sounds as measured by the mismatch negativity (MMN) between 100 and 250 ms in contrast to their typically developing peers (Froyen et al., 2009). Another study found (Žarić et al., 2014) that in an audiovisual oddball task deviant vowels elicited typical mismatch responses in the auditory condition even in 9-year-old children with dyslexia; however, the mismatch responses were reduced in the AV condition. In fact, children with severe dyslexia showed a small mismatch effect in the N1 time window, while less dysfluent and typical readers showed a mismatch effect in both the N1 and P2 time windows. In addition, the latency of the MMN response was related to individual differences in reading fluency indicating impairment in grapheme-phoneme mapping. Furthermore, Žarić et al. (2015) demonstrated that the MMN latency is also related to reading gains after an extensive letter-speech sound mapping training providing further evidence for the role of deficient orthographic-phonological integration in dysfluent reading.

Although several studies provide insight into the deficits of AV integration of single letters, less is known about the AV integration of letter strings and spoken words. To investigate the latter, Kronschnabel et al. (2014) tested the audiovisual integration deficit in dyslexia by presenting congruent and incongruent three-letter audiovisual stimuli in an implicit target detection task. Although the EEG data did not reveal group differences in audiovisual integration, fMRI data indicated impaired processing of audiovisual stimuli. Moreover, despite no group differences were found during single letter processing in the EEG data, the AV integration deficit was pronounced for three-letter long strings indicating specific deficits in processing word-like stimuli (see also Mittag et al., 2013).

Moreover, a study by Jost et al. (2014) tested AV integration by presenting first-grade readers with familiar German or unfamiliar English written words along with congruent (identical) and incongruent (all letter different) auditory words. Children showed a congruency effect but only for familiar German words suggesting that the effect is modulated by lexical-semantic information. To advance results on audiovisual processing of written words, Wang et al. (2020) presented first-grade readers with congruent and incongruent audiovisual pseudowords in their fMRI study. Children did not show a congruency effect in first grade, but when re-measured in second-grade, a congruency effect emerged, and the development of the effect was related to the pseudoword reading fluency.

While it seems that both audiovisual and visual/orthographic processing can be deficient in developmental dyslexia, the relationship between the two processes should be considered, as well. As McCandliss et al. (2003) argue, the development of brain areas responsible for multimodal integration modulates the tuning of visual areas for print. In addition, the phonological mapping hypothesis (Maurer et al., 2007) also proposes that the left hemispheric lateralization of word N1 is due to automatized grapheme-phoneme integration. In line with this, several studies found association between grapheme-phoneme mapping and visual sensitivity for print. For instance, Brem et al. (2018) demonstrated that sensitivity for a novel script emerges in a two h character-sound association training regardless whether novel visual stimuli are trained with spoken syllables or spoken words which suggests that training related modulation of the visual N1 is due to phonological associations. Furthermore, in a series of experiments, Maurer and colleagues (Maurer et al., 2010; Yoncheva et al., 2010, 2015) explicitly compared the modulation of print N1 after a grapheme-phoneme focused training and a whole word focused training. The grapheme-phoneme mapping training resulted in left-lateralized N1 response whereas the whole word training resulted in right-lateralized N1 response. Even more interestingly, Fraga-González et al. (2017) reported that audiovisual integration as indexed by the MMN latency was correlated with changes in the visual N1 for words after reading fluency training in children with dyslexia. The result suggests that the severity of audiovisual integration deficit and the level of visual sensitivity for print are related and together with the above results provides further evidence that audiovisual integration might modulate print sensitivity.

Lastly, Hasko et al. (2012) explicitly tested the contribution of orthographic processing deficit and audiovisual integration deficit to reading disfluency by comparing the ERP responses of children with and without dyslexia in a visual-visual and an auditory-visual condition. The researchers found that 11 years old children with developmental dyslexia showed different N300 responses compared to control children for stimuli requiring orthographic-phonological mapping. In addition, the N300 response correlated with reading fluency. However, the groups did not differ in processing visual stimuli, which only requires orthographic processing. Similarly, children with and without dyslexia did not differ in their N170 responses, which suggests that reading deficits in dyslexia might be traced to inefficient integration of orthographic and phonological information rather than orthographic processing deficits. Nonetheless, the above study used only real words as stimuli and the task was confounded with phonological working memory skills since children had to hold the auditory reference stimulus in memory to be able to compare it the visual target stimulus.

In the current study, we aim to explore audiovisual processing of orthographic stimuli by adult readers with and without dyslexia in an implicit same-different (perceptual matching) task. Experimental paradigms used previously (such as the one-back task or the explicit same-different perceptual-matching task) are often confounded by working memory and attentional factors because participants are required to pay attention to differences between the stimulus pair. Therefore, differences between participants with and without dyslexia can result from differences in memory skills or attentional span. In our implicit same-different task, memory bias is excluded by analyzing the N1 responses to the reference stimuli and by presenting the auditory and visual stimuli concurrently in the audiovisual condition. Moreover, participants were instructed to indicate when a stimulus appeared in bold fonts; thus, the paradigm does not require reading. Furthermore, our paradigm made it possible to explore the main processes that were found to be inefficient in developmental dyslexia (fine-grade print sensitivity as indexed by the lexicality effect, orthographic coding as indexed letter identity and position coding, and audiovisual integration) in one single study.

First, we investigated whether (1) orthographic processing deficits are present in dyslexia for both words and pseudowords. Since previous studies demonstrated a lexicality effect on N1 in skilled readers but not in readers with dyslexia, we expected to find differential N1 response modulation as a function of lexicality and reading skill. Then, we examined whether (2) inefficient orthographic processing could be traced by measuring decoding of letter identity and position. To this end, participants were shown stimulus pairs that could be either identical (ID), different in the identity of one letter (letter identity neighbor, IN), or different in the position of the letters (letter position pairs, PP). Previous studies reported deficits in letter identity and position processing in readers with dyslexia; therefore, we expected to find differential effect of the pair type on the N1, N250 as a function of reading skill. Finally, to explore whether (3) orthographic-phonological processing is more deficient than orthographic processing in dyslexia, target stimuli were presented in visual-only and audiovisual conditions. We expected to find differential effects of lexicality and pair type as a function of reading skill and modality already on the N1 and N250 components. More specifically, we expected that group differences would be greater in the audiovisual condition compared to the visual only condition.

## Materials and Methods

### Participants

Twenty-seven high functioning readers with dyslexia (DL) and 31 control readers (CL) participated in the experiment; however, two participants from the dyslexia group and four participants from the control group were excluded from the analysis due to low numbers of accepted trials per condition (see details in the EEG recording and data preprocessing section). Finally, 25 participants with dyslexia [10 female, mean age 21.12 years, *SD* = 3.78, range = 18–34 years, five left-handed according to the Edinburgh Handedness Inventory (Oldfield, 1971)] and 27 control participants (15 female, mean age 21.89 years, *SD* = 2.89, range = 18–28 years, all right handed) were included in the analysis. All participants were native Hungarian speakers and had normal or corrected-to-normal vision and intact hearing according to the screening audiometry (250–8,000 Hz). Participants with dyslexia were recruited through advertisements. All of them had been diagnosed with dyslexia during childhood and completed remediation training with a speech therapist. None of the participants except for one had a clinical diagnosis of ADHD. Control participants had no history of reading disorders. Participants' informed consent was obtained in written form from all participants, and the experimental protocol was approved by the United Ethical Review Committee for Research in Psychology.

### Individual Differences Measures

Prior to the EEG experiment, the reading-related skills of all participants were assessed through the Hungarian version of the Differential Diagnosis Dyslexia Battery (Tóth et al., 2014). Reading fluency was measured by three subtasks: high-frequency word reading, low-frequency word reading, and pseudoword reading. The *reading fluency* score was calculated from the three subtasks as the correctly read items per second. The *reading accuracy* score was calculated from the three subtasks as the correctly read items. In addition, *rapid automatized naming (RAN)* with letters, digits, and objects and the *phoneme deletion* were measured, as well.

In addition, we measured s*entence reading fluency* with an in-house task in which participants read a list of 40 sentences and indicated whether the sentence's meaning is true or false. As the sentences are semantically very simple; reading speed is determined by word reading fluency rather than reading comprehension. Thus, the sentence reading fluency score was calculated as the mean log reaction time for correctly answered sentences.

To assess orthographic knowledge, participants were presented with a list of 42 sentences in a *proofreading* task. They were instructed to quickly click on the misspelled word with the mouse in every sentence. The misspellings were of three types: (1) two letter were transposed (TL), (2) one letter was substituted with another letter from the alphabet (SL1), (3) two letters were substituted with another letter from the alphabet (SL2). The proofreading score was calculated as the mean log reaction time for the correctly identified misspelled words.

*Spelling* was measured with an in-house multiple choice spelling test. Altogether 44 items were presented; participants used the mouse to indicate their response. Two scores were calculated: spelling accuracy (mean correct percent of responses) and spelling reaction time (mean log RT). Descriptive statistics for the groups are presented in Table 1.

**Table 1:** Descriptive statistics of participants with and without dyslexia and group differences (*t*-test).

	**Dyslexia (*****n*** **= 25)**		**Control (*****n*** **= 27)**		*t*-value^*^
	**Mean**	**SD**	**Mean**	**SD**	
Age (years)	21.12	3.78	21.89	2.89	−0.82
Reading Fluency (item/s)	0.76	0.32	1.27	0.31	−5.84^***^
Reading accuracy (%)	93.16	6.38	97.36	3.3	−2.94
RAN Letter (item/s)	2.18	0.41	2.60	0.38	−3.85^**^
RAN Number (item/s)	2.37	0.46	2.90	0.45	−4.21^**^
RAN Object (item/s)	1.57	0.24	1.72	0.28	−2.09
Phoneme deletion accuracy (%)	86.23	13.59	96.99	4.82	−3.75^**^
Phoneme deletion speed	7.85	0.35	7.41	0.27	5.03^***^
Sentence reading fluency^a^	8.06	0.34	7.60	0.21	5.92^***^
Proofreading^b^	8.45	0.43	7.64	0.27	7.97^***^
Spelling accuracy (%)	47.01	7.35	62.98	12.41	−5.62^***^
Spelling speed	8.19	0.3	7.73	0.21	6.34^***^

*Accuracy scores are presented as percent correct. Speed measures are expressed as log reaction times. Reading Fluency is expressed as the correctly read items per second; therefore, higher fluency score indexes faster reading*.

a *Data is missing for one participant*.

b *Data is missing for three participants*.

* *p < 0.05*,

** *p < 0.01*,

*** *p < 0.001 after Bonferroni correction*.

### Stimuli

In the EEG session, we employed an implicit same-different task which included blocks of word, pseudoword, character, and digit stimuli. Here we focus on words and pseudowords because only these stimuli were presented both in a visual and an audiovisual condition. Thus, two types of stimuli were used: 360 word pairs and 360 pseudoword pairs.

One hundred and eight base words were selected from the Hungarian National Corpus (HNC, Váradi, 2002) that had two different word pairs: (1) a word that differed in the position of the letters and (2) a word that differed in the identity of one letter. Thus, the reference-target pairs could be either identical (ID, e.g., MANGÓ-MANGÓ [mango]), or different in one substituted letter (letter identity neighbor, IN, e.g., MANGÓ-MARGÓ [mango-margin]), or different in the position of their letters (letter position pairs, PP, e.g., MANGÓ-MAGNÓ [mango-tape recorder]). The words were mono- and bisyllabic and did not contain digraphs or trigraphs. Mean log bigram frequency (and standard deviation) of the base words was 13.64 (1.33). In addition, 12 word triplets were selected to serve as filler items. This resulted in 360 word pairs among which 120 were three letters, 120 were four letters, and 120 were five letters long.

In addition, 108 pseudowords triplets of 3–5 letter length were created to match the word triplets. From each of the base words described above, three pseudowords were created by changing letters in the base word. The resulting pseudowords were not part of the HNC or the CELEX database. The pseudowords could be either identical (ID, e.g., ZONAT-ZONAT—from the base word “vonat” [train]), different in one substituted letter (IN, e.g., ZONAT-BONAT), or different in the position of their letters (PP, e.g., ZONAT-TAZON). Mean log bigram frequency (and standard deviation) of the pseudowords was 13.24 (1.47). In addition, 12 pseudoword triplets were created to serve as filler items. This resulted in 360 pseudoword pairs altogether (120 were three letter, 120 were four letters, and 120 were five letters long).

The word pairs and pseudoword pairs were also presented in an audiovisual condition. Thus, the target stimuli were presented visually together with an auditory stimulus. The auditory stimuli were digitally recorded from a male native Hungarian speaker in a soundproof room (sampling rate was 44.1 kHz presented to both ears via headphones (AKG K401) with an intensity of approximately 75 dB. Sound duration was 697 ms (*SD* = 112.96 ms, range = 444.81−1052.15 ms) for words and 683 ms (*SD* = 106.97 ms, range = 431.81−1040.54 ms). The auditory stimuli were identical to the visually presented target stimuli; therefore, for the IN and PP pairs, there was a mismatch between the visual reference and visual target and the visual reference and auditory target. The number of graphemes and phonemes in the visual and auditory stimulus were always identical. To counterbalance the stimulus presentation across conditions, two stimulus lists were prepared. In each list, half of the word (180 pairs) and half of the pseudoword (180) pairs were presented visually whereas the other half of words (180 pairs) and pseudoword (180) pairs were presented audiovisually. Thus, the lists equated lexicality (w/pw) and modality of presentation (V/AV).

Overall, 720 stimulus pairs were presented: 648 reference-target pairs and 72 filler pairs. The reference stimuli were always presented only visually, while half of the target stimuli were presented only visually (V), and the other half was presented audio-visually (AV). Stimuli were presented in 12 separate blocks according to modality (V/AV), lexicality (w/pw) and length (3/4/5 letter long). Order of the word and pseudoword blocks were randomized across participants. Visual blocks always preceded audiovisual blocks in order to avoid carry-over effects from the enhanced grapheme-phoneme mapping in the AV condition.

In each block, there were 60 stimulus pairs among which 54 were reference—target pairs and six were filler pairs. Filler items accounted for 10% of all trials and were not included in the analysis. The number of identical, transposed-letter, and substituted-letter trials was balanced within and across blocks. The order of stimuli was randomized within and across blocks. The full stimulus list is presented in Supplementary Table 1.

### Procedure

During the EEG experiment, participants were individually tested in a soundproof, electrically shielded room. Stimulus presentation and response recording was carried out with Presentation 15.1 software; all stimuli were presented on a 22” LED computer screen with a refresh rate of 60 Hz positioned at a distance of 70 cm from the participants. The target stimulus of the filler pairs was presented in bold, and participants were required to indicate the appearance of these items by pressing a button. Before the first block of word and first block of pseudoword stimuli, there were four practice trials among which one was presented in bold. All stimuli were presented in black capital letters in DejaVu Sans Mono font on a blue-gray background. Reference stimuli were 28 font-size, whereas target stimuli were 32 font-size; targets of filler pairs were presented in bold fonts.

**Figure 1:**
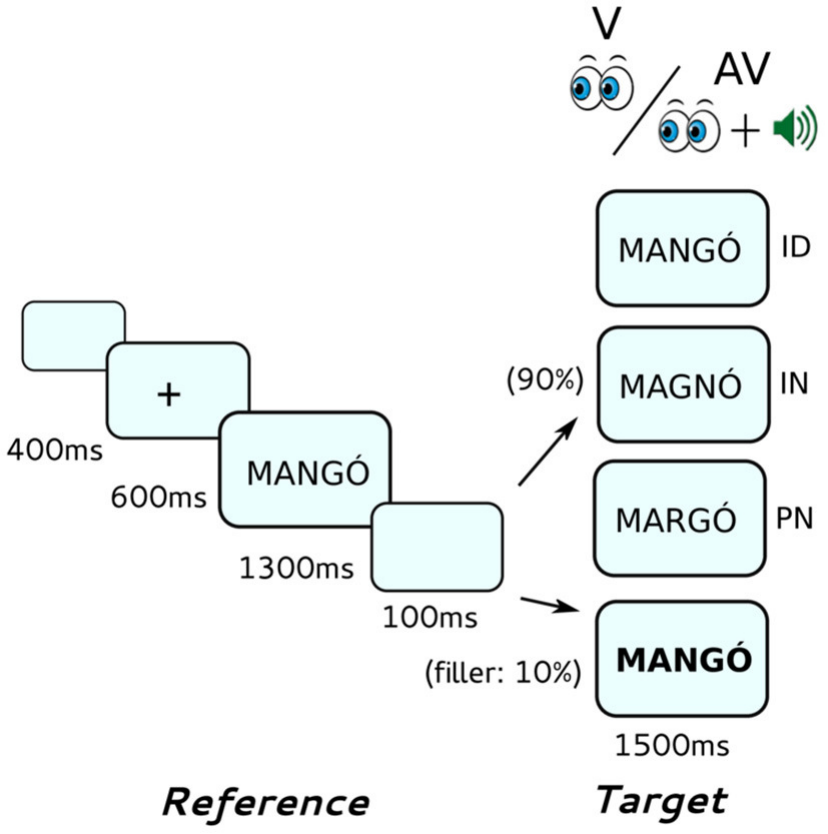
Outline of the same-different implicit reading task. Reference stimuli were always presented visually, target stimuli were presented either visually only (V) or audiovisually (AV). Target stimuli could be either identical (ID), different in the identity of one letter (IN) or different in the position of letters (PP) compared to the reference stimuli.

Each trial (see Figure 1) started with a blank screen displayed for 400 ms followed by a 24 font-size fixation cross at the middle of the screen for 600 ms. Then, the reference stimulus was displayed for 1,300 ms followed by a blank screen for 100 ms. Finally, the target stimulus was displayed for 1,500 ms. In the AV condition, an auditory target stimulus was also presented synchronized to the onset of the visual target stimulus.

### EEG Recording and Data Preprocessing

Data was recorded with a 32-channel Easy Cap (EASYCAP GmbH, Herrsching, Germany), electrodes were positioned according to the international 10–20 system guidelines. The EEG was recorded continuously with a Cz reference, a 1,000 Hz/channel sampling rate, and a 0.01–100 Hz bandpass filter. Electrode impedances were kept below 10 kΩ. Data preprocessing was performed with Brain Vision 2.0 software (Brain Products GmbH). First, data was filtered (0.1−30 Hz zero-phase Butterworth IIR bandpass filter, 24 dB/oct). Next, eye movements were corrected with ICA (Jung et al., 2000). The mean number of ICs and standard deviations (in parenthesis) corrected were 3.11 (1.89) for the control group and 3.6 (1.85) for the group with dyslexia. Then, data were baseline corrected (100 ms prior stimulus presentation), segmented into 600 ms epochs, and re-referenced to average reference (Lehmann and Skrandies, 1980). Finally, trials containing artifacts exceeding +/– 200 μV were rejected; the maximum-minimum voltage difference was 200 μV. In each condition, at least 38 (out of 54) artifact-free trials were required to include a participant in the analysis. Mean trial numbers and standard deviations (in parenthesis) were as following: word reference stimuli: 52.89 (1.88) for CL, 52.54 (2.09) for DL; pseudoword reference stimuli: 52.87 (1.77) for CL and 52.63 (1.99) for DL; visual word targets: 52.56 (2.08) for CL [ID: 52.44 (1.72), IN: 52.81 (2.27), PP: 52.41 (2.26)] and 52.88 (1.68) for DL [ID: 53.2 (1.35), IN: 52.72 (2.19), PP: 52.72 (1.37)]; visual pseudoword targets: 52.37 (2.84) for CL [ID: 52.58 (2.36), IN: 52.30 (3.18), PP: 52.33 (3.03)] and 52.35 (2.58) for DL [ID: 51.52 (3.38), IN: 52.6 (2.14), PP: 52.92 (1.85)]; audiovisual word targets: 52.90 (1.74) for CL [ID: 52.96 (2.23), IN: 52.78 (1.67), PP: 52.96 (1.26)] and 52.47 (2.40) for DL [ID: 52.16 (3.45), IN: 52.68 (1.68), PP: 52.56 (1.71)]; audiovisual pseudoword targets: 52.93 (2.05) for CL [ID: 52.82 (2.32), IN: 53.19 (2.24), PP: 52.78 (1.58)] and 52.43 (2.39) for DL [ID: 52.4 (2.30), IN: 52.36 (2.63), PP: 52.52 (1.69)]. The acceptable trials were averaged for participants and conditions.

### Data Analysis

Data analysis was carried out using the eegR package (Tóth, 2015) available in the R environment (R Core Team, 2013). To assess the processing of word and pseudoword pairs, a Topographic Analysis of Variance (TANOVA, Lehmann and Skrandies, 1980; Strik et al., 1998) on ERP maps was computed for each time point. This approach treats ERP data as a sequence of ERP maps changing in topography and strength over time (Lehmann and Skrandies, 1980) and is sensitive to differences at particular electrodes without specifying them. ERP map strength can be characterized by the Global Field Power (GFP), which is the standard deviation of the potentials at all electrodes of an average-reference map. ERP map topography can be calculated as the difference of normalized maps (global map dissimilarities, GMD). While TANOVA on raw maps detects all systematic amplitude (GFP) differences between the maps, TANOVA on normalized maps detects only topographic differences (GMD).

In our data analysis, we ran point-to-point TANOVAs to determine whether experimental effects are due to differences in intensity (GFP) or topography (GMD). We computed GFP and GMD for each time-point, created a probability distribution (with *n* = 4,999 L permutation in order to control for multiple comparisons, permuted *p*-value, *P*_perm_), and calculated a z-score of the original dissimilarity. We report the median values of z-scores and permuted *p*-values for those data points, which were significant at the level of 0.05.

ERP data were analyzed separately for reference and target stimuli. Reference stimuli were analyzed in a repeated measure point-to-point TANOVA with lexicality (w/pw) as a within-subject factor and with group (DL/CL) as a between-subject factor. Target stimuli were analyzed with lexicality (w/pw), modality (V/AV), and pair type (ID/IN/PP) as within-subject factors and with group (DL/CL) as a between-subject factor. To compare the effects in the different modalities, we also ran analysis for the V and AV conditions separately.

To ease the comparison of our results with previous studies, we also performed traditional ERP analyses. Adaptive segmentation based on the GFP minima (Maurer et al., 2005b) was done for the grand averaged means. GFP was calculated separately for the reference and target pairs for adults with and without dyslexia in the time range of 0–600 ms (averaged for lexicality, pair type, and modality).

For the reference stimuli, we used repeated measures ANOVA with group (DL/CL) as between- subject factors, whereas lexicality (w/pw) and laterality (left/right) served as within- subject factors. According to the GFP segmentation, in the N1 segment, control readers exhibited the most activity at 138–337 ms (peak: 227 ms), whereas participants with dyslexia had greater activity at 140–276 ms (peak: 215 ms). Since the segmentation resulted in similar time windows for the groups, the rest of the analysis will use the general segmentation of the stimuli (139–324 ms, peak: 223 ms).

**Figure 2:**
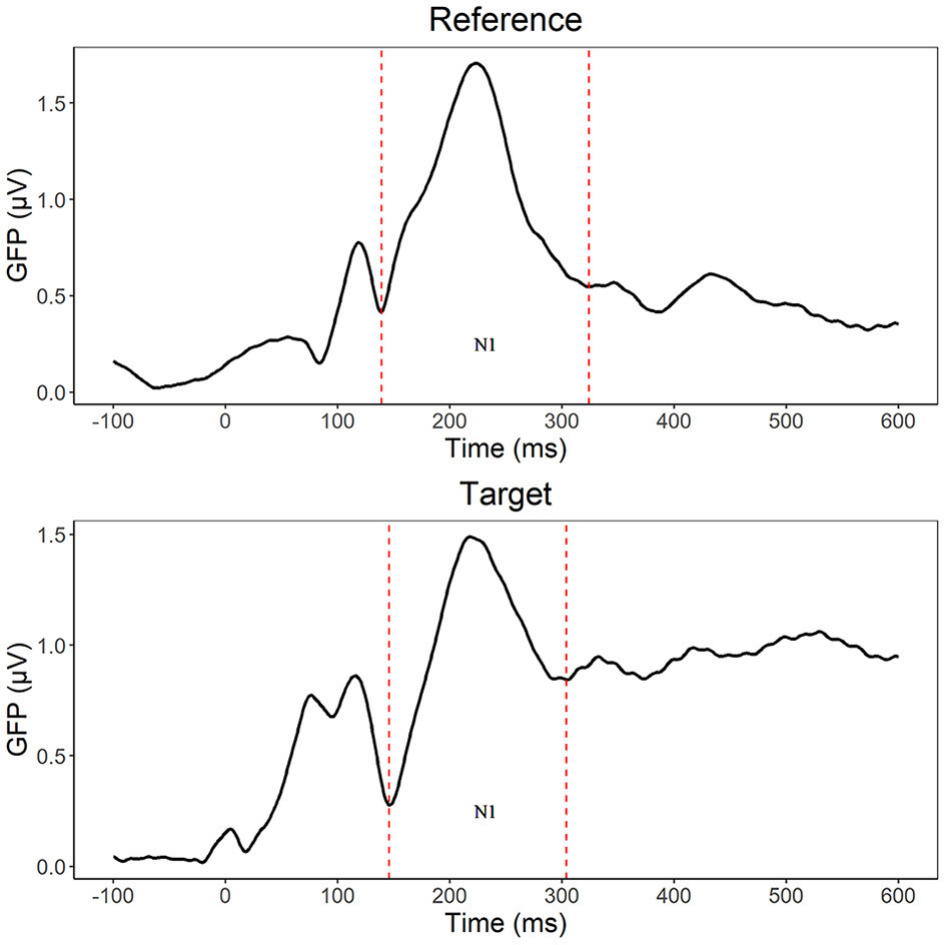
N1 segmentation (dashed line) based on Global Field Power (GFP) separately for reference (139–324 ms) and target (146–304 ms) stimuli.

For the target stimuli, we also used repeated measures ANOVA with the same factors as in the analysis of reference stimuli, but also added the within- subject factors pair type (ID/IN/PP) and modality (AV/V). According to the GFP segmentation (see Figure 2), the GFP window for control group is at 147–305 ms (peak: 218 ms), whereas the time window for the group with dyslexia is at 146–290 ms (peak: 218 ms). Again, analysis will use the general segmentation of the stimuli (146–304 ms, peak: 218 ms, V targets: 140–304 ms, peak: 231 ms, AV targets: 154–292 ms, peak: 216 ms). For the letter identity and position encoding analyses, aside from the occipito-temporal sites used in the reference stimuli, we used channel clusters from the frontal-central channels (F3, P3, C3, Fz, Cz, Pz, F4, P4, C4 based on Duñabeitia et al. (2009). Lastly, we used the Greenhouse-Geiser correction to adjust critical *p* values when the assumption of sphericity is violated.

## Results

### Behavioral Results

Filler items were included only to maintain attention during the experiment; thus, responses for these items were excluded from the EEG analysis. Mean hit rates were 0.999 (*SD* = 0.001) for the dyslexia and 0.996 (*SD* = 0.008) for the control group.

### Lexicality Effect

To compare whether lexicality (word and pseudoword processing) effect occurred for both groups, we analyzed the reference stimuli (which were always presented visually).

In the GFP analysis, no effect reached the significance level (group: z-score = 0.96, *P*_perm_ = 0.668; lexicality: z-score = 0.69, *P*_perm_ = 0.487; group x lexicality: z-score = 0.51, *P*_perm_ = 0.491).

**Figure 3:**
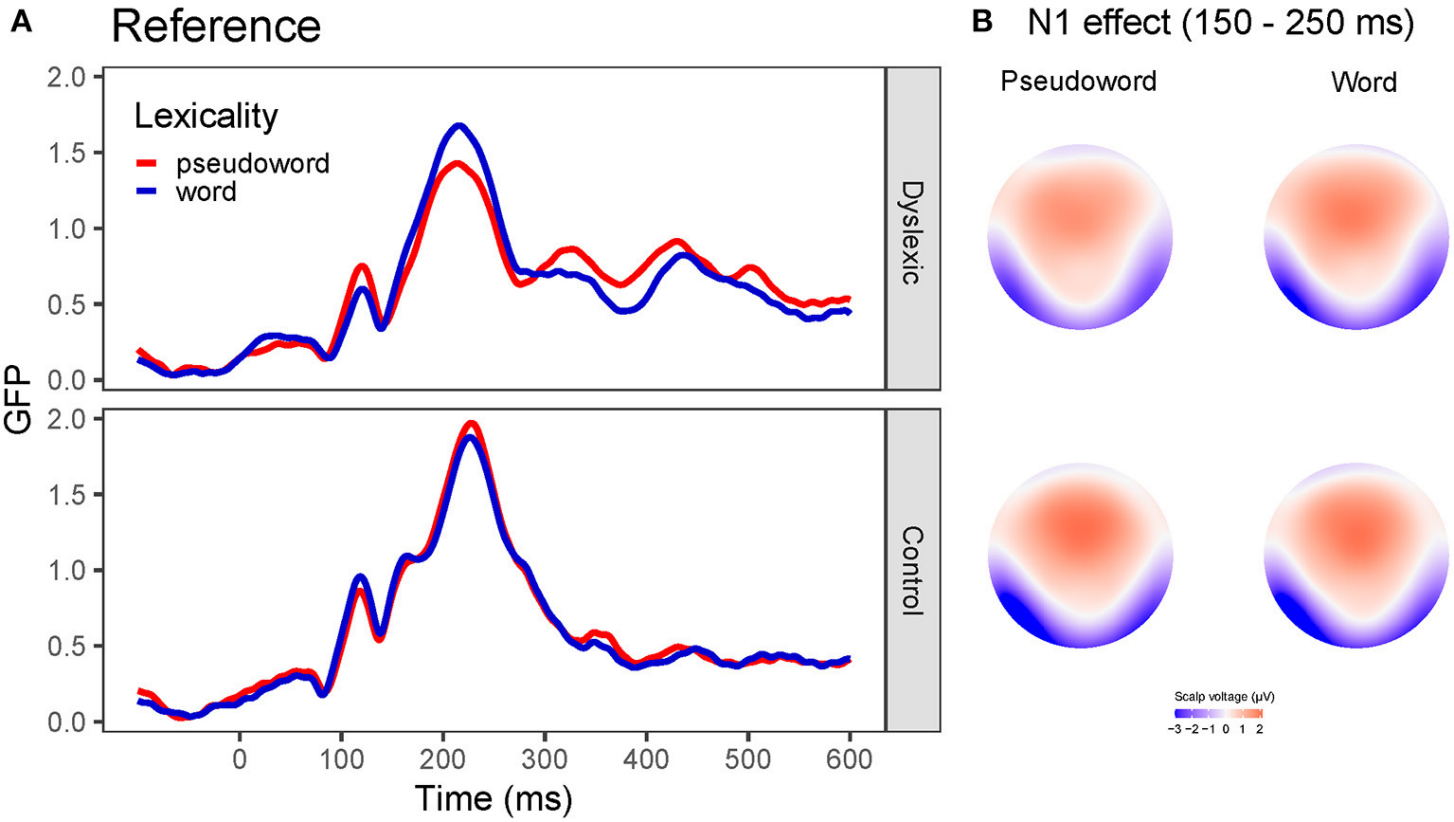
Global field power (GFP) curves and topographic maps for the reference stimuli. Note that reference stimuli were always presented visually. **(A)** GFP for the word and pseudoword stimuli separately for the dyslexia and control group. **(B)** Topographic maps for the N1 effect for the word and pseudoword stimuli separately for the groups with and without dyslexia averaged between 150 and 250 ms.

According to the GMD analysis, topography did not differ as a function of lexicality (z-score = 0.67, *P*_perm_ = 0.416) or group (z-score = 1.37, *P*_perm_ = 0.101). The group x lexicality interaction did not reach the significance level (z-score = 0.76, *P*_perm_ = 0.574). GFP curves and topographic maps are presented on Figure 3.

**Figure 4:**
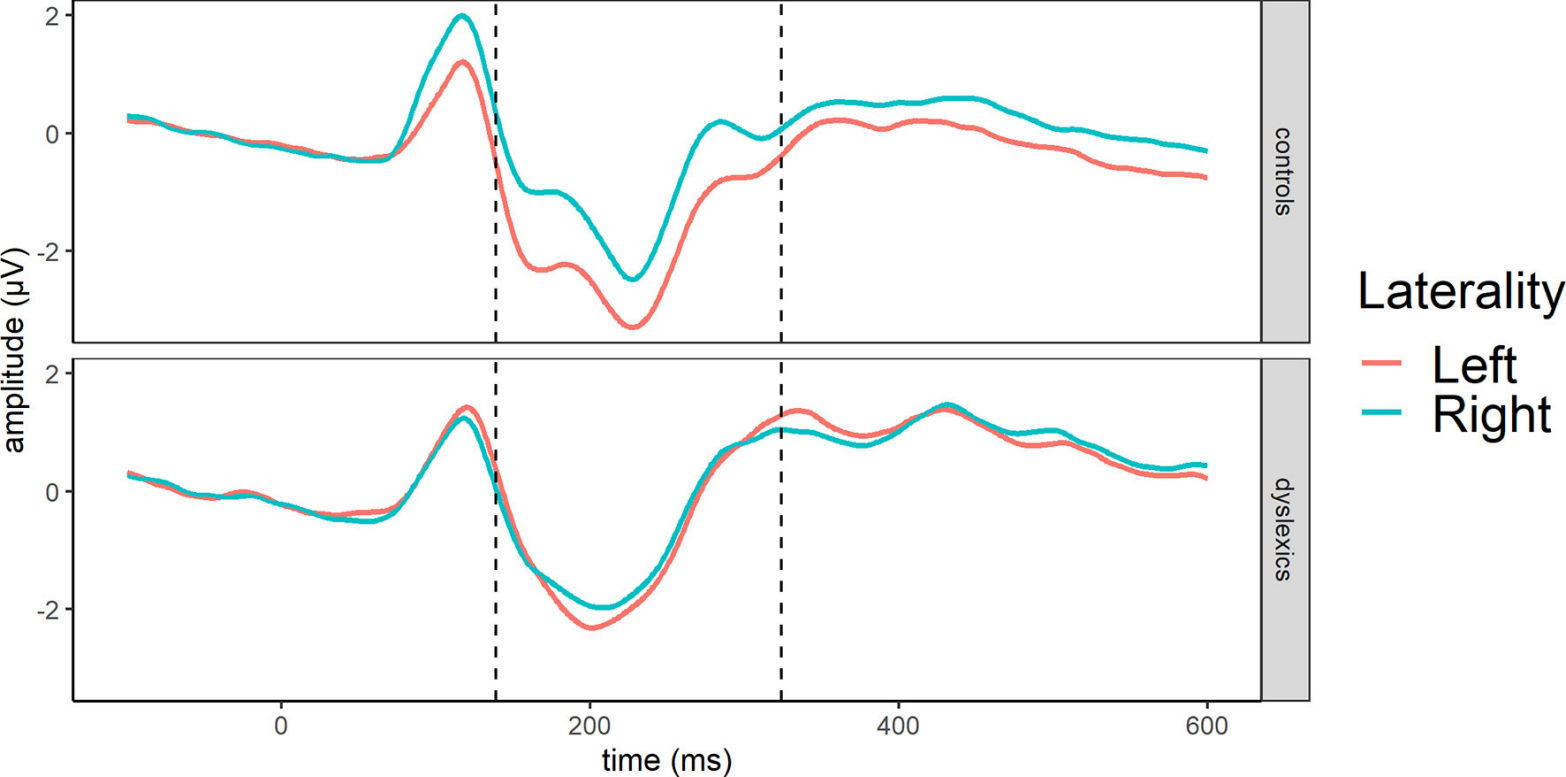
Averages across occipito-temporal electrodes (O1, O2, P7, P8) for N1 in reference stimuli showing group and laterality interaction at GFP-based N1 segment (dashed line): 139–324 ms.

The traditional analysis in the reference stimuli (139–324 ms) showed a significant effect of laterality [*F*_(1, 50)_ = 9.80, *p* = 0.003, $\eta_{g}^{2}$ = 0.03] while the main effects of group [*F*_(1, 50)_ = 2.61, *p* = 0.113, $\eta_{g}^{2}$ = 0.04] or lexicality [*F*_(1, 50)_ = 0.95, *p* = 0.334, $\eta_{g}^{2}$ = 0.001] were not significant. In addition, laterality interacted with group [*F*_(1, 50)_ = 5.82, *p* = 0.020, $\eta_{g}^{2}$ = 0.02] (Figure 4) since a significant effect of laterality was observed only in controls [*F*_(1, 26)_ = 14.04, *p* < 0.001, $\eta_{g}^{2}$ = 0.08] due to greater responses in the left hemisphere, whereas the participants with dyslexia did not show any difference between the hemispheres [*F*_(1, 24)_ = 0.21, *p* = 0.651, $\eta_{g}^{2}$ = 0.002]. Meanwhile, group x lexicality [*F*_(1, 50)_ = 3.27, *p* =0.0766, $\eta_{g}^{2}$ = 0.004], lexicality x laterality [*F*_(1, 50)_ = 0.03, *p* = 0.870, $\eta_{g}^{2}$ = 0.000002], or group x lexicality x laterality interaction [*F*_(1, 50)_ = 0.13, *p* =0.720, $\eta_{g}^{2}$ = 0.00001] did not reach significance.

In sum, controls exhibited a more left-lateralized N1 compared to the group with dyslexia. Nevertheless, words and pseudowords are processed similarly, and readers with and without dyslexia do not differ in processing orthographic stimuli in the N1 time window.

### Letter Identity and Position Encoding

To test whether readers with dyslexia process letter identity and position inefficiently, we analyzed the pair type effect (ID/IN/PP), the lexicality effect (w/pw), and the group effect (DL/CL) in the visual targets.

In the GFP analysis, the main effect of group (z-score = 3.31, *P*_perm_ = 0.004) between 185 and 380 and 493 and 600 ms indicated that controls showed greater responses than readers with dyslexia. The main effect of lexicality (z-score = 3.46, *P*_perm_ = 0.005) between 311 and 400 ms was present as word stimuli evoked greater responses than pseudoword stimuli. There was also a main effect of pair type (z-score = 3.11, *P*_perm_ = 0.005) between 144 and 235 ms and 455 and 600 ms. Based on visual inspection and the traditional analysis below, in the early time-window (144–235 ms), ID targets evoked smaller responses compared to PP or IN pairs. In the later time window (455–600 ms), IN targets evoked smaller responses than ID or PP targets. This was the case for the participants both with and without dyslexia signified by the lack of significant interactions (group x lexicality: z-score = 0.82, *P*_perm_ = 0.416, group x pair type: z-score = 0.61, *P*_perm_ = 0.609, lexicality x pair type: z-score = 0.62, *P*_perm_ = 0.550, group x lexicality x pair type: z-score = 0.67, *P*_perm_ = 0.545).

**Figure 5:**
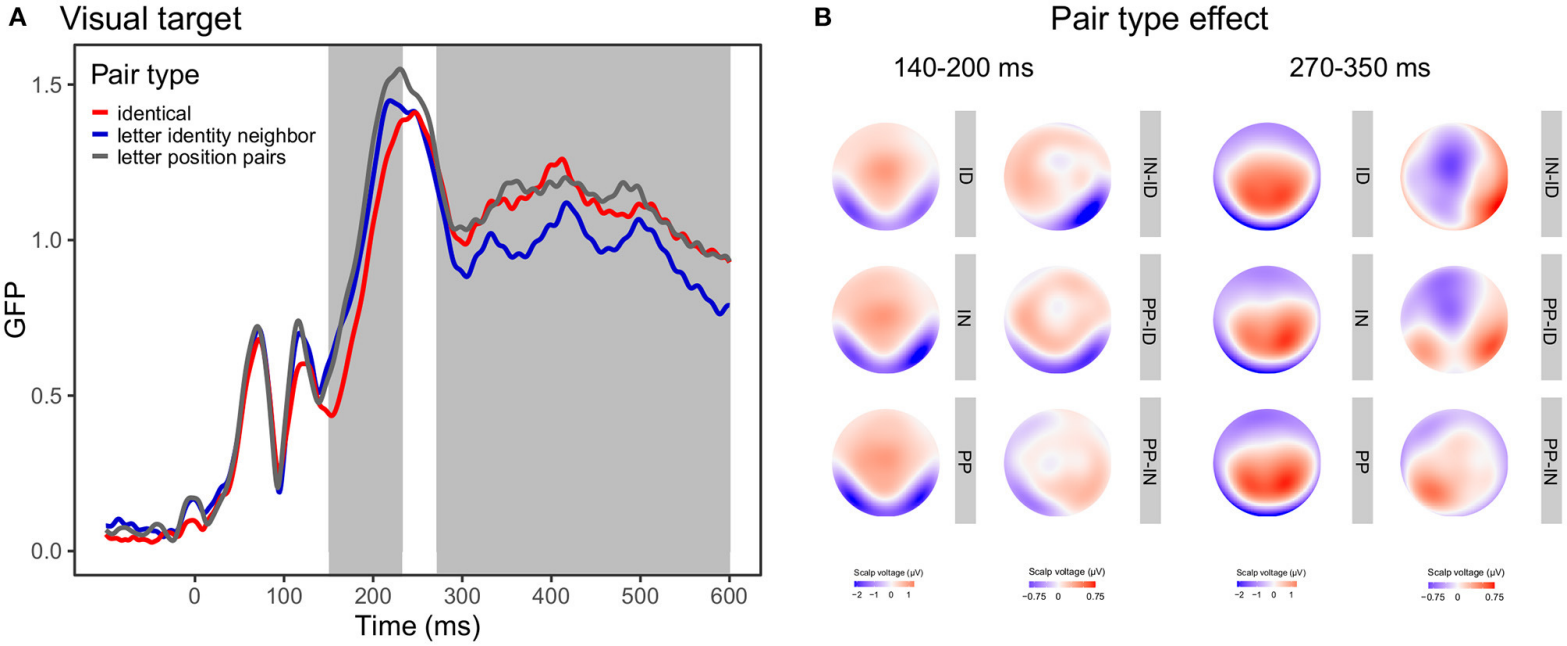
Global field power (GFP) curves and topographic maps for the pair type effect in visual target stimuli. **(A)** GFP for the identical, letter identity neighbor, and letter position pair targets. Gray areas highlight the time windows in which the effect was significant according to the GMD analysis. **(B)** Topographic maps for the pair type conditions (ID, identical; IN, letter identity neighbor; PP, letter position pairs) and the pair type differences averaged between 140-200 ms representing the first time window and between 270–350 ms representing the second time window.

In the GMD analysis, the main effect of group (z-score = 4.49, *P*_perm_ < 0.001) between 255 and 600 ms and the main effect of pair type (z-score = 3.03, *P*_perm_ = 0.006) between 89 and 129, 142 and 206 and 273 and 600 ms was significant. Group effect was present because controls exhibited more left-lateralized responses than readers with dyslexia. Pair type effect in the early time window (142 and 206 ms) resulted from the different scalp topographies of ID vs. IN. Pair type effect in the late time window (273 and 600 ms) resulted from the different scalp topographies of ID vs. IN, ID vs. PP, and IN vs. PP pairs. No other effects were significant at any time point (lexicality: z-score = 0.76, *P*_perm_ = 0.443; group x lexicality: z-score = 0.57, *P*_perm_ = 0.467; group x pair type: z-score = 0.56, *P*_perm_ = 0.525; lexicality x pair type: z-score = 0.78, *P*_perm_ = 0.579; group x lexicality x pair type: z-score = 0.71, *P*_perm_ = 0.665). Topographic maps and GFP curves for the pair type effect are presented on Figure 5.

The traditional analysis on the N1 segment on occipital-temporal sites for visual targets (140–304 ms, peak: 231 ms) revealed a significant effect on group [*F*_(1, 50)_ = 6.81, *p* = 0.012, $\eta_{g}^{2}$ = 0.08], wherein control participants showed more negative responses than participants with dyslexia. Moreover, the interaction between lexicality and laterality [*F*_(1, 50)_ = 5.23, *p* = 0.027, $\eta_{g}^{2}$ = 0.001] was also significant. The interaction was present as word targets were somewhat more left-lateralized than pseudoword targets, though laterality was not significant for either the words [*F*_(1, 50)_ = 3.34, *p* =0.073, $\eta_{g}^{2}$ = 0.01] or the pseudowords [*F*_(1, 50)_ = 0.34, *p* = 0.561, $\eta_{g}^{2}$ = 0.0008] when analyzed separately. The main effect of pair type was marginally significant [*F*_(2, 100)_ = 3.04, *p* = 0.0525, $\eta_{g}^{2}$ = 0.004]. The pair type effect suggested that ID targets elicited somewhat less negative response than PP (*p* = 0.15, *p*_*bonferroni*_ = 0.44) or IN (*p* = 0.20, *p*_*bonferroni*_ = 0.61), while PP and IN did not differ from each other (*p* = 0.85, *p*_*bonferroni*_ = 1.0). No other effects or interactions were significant [see (2) Letter identity and position encoding section in the Supplementary Material].

On the selected frontal-central sites, repeated measures ANOVA showed significant main effects on group [*F*_(1, 50)_ = 8.53, *p* =0.005, $\eta_{g}^{2}$ = 0.10], in which controls readers generated a bigger response than readers with dyslexia. In addition, the pair type effect was marginally significant [*F*_(2, 100)_ = 3.02, *p* =0.053, $\eta_{g}^{2}$ = 0.005]. Furthermore, neither the lexicality effect [*F*_(1, 50)_ = 0.50, *p* = 0.485, $\eta_{g}^{2}$ = 0.001] nor the interactions [see (2) Letter identity and position encoding section in the Supplementary Material] showed any significant effect.

In sum, the group effect did not interact with either the lexicality or the pair type effect suggesting similar processing of orthographic stimuli in the visual modality despite topographic differences between the groups.

### Audiovisual Processing

To test the audiovisual processing deficits in dyslexia, we compared the processing of target stimuli in the visual and audiovisual conditions.

#### Visual Condition

As described in the *Letter identity and position encoding* section, for visual targets there was a main effect of group (GFP: z-score = 3.31, *P*_perm_ = 0.004, 185–380 and 493–600 ms, GMD: z-score = 4.49, *P*_perm_ < 0.001, 255–600 ms), a main effect of lexicality (GFP: z-score = 3.46, *P*_perm_ = 0.005, 311–400 ms), and a main effect of pair type (GFP: z-score = 3.11, *P*_perm_ = 0.005, 144–235 ms and 455–600 ms, GMD: z-score = 3.03, *P*_perm_ = 0.006, 89–129, 142–206 and 273–600 ms), but the interactions were not significant indicating that readers both with and without dyslexia process orthographic stimuli similarly in the visual modality. Similar results emerged from the traditional analysis showing a main effect of group [*F*_(1, 50)_ = 6.81, *p* = 0.012, $\eta_{g}^{2}$ = 0.08], a lateralized lexicality effect [lexicality x laterality: *F*_(1, 50)_ = 5.23, *p* = 0.027, $\eta_{g}^{2}$ = 0.001], and a marginal effect of pair type [*F*_(2, 100)_ = 3.04, *p* = 0.053, $\eta_{g}^{2}$ = 0.004].

#### Audiovisual Condition

As opposed to this, for the audiovisual targets, GFP analysis revealed a group main effect (z-score = 3.82, *P*_perm_ = 0.001, 190–379 ms) due to greater responses of readers without dyslexia compared to readers with dyslexia. The group x lexicality interaction (z-score = 2.35, *P*_perm_ =0.027) between 178 and 218 ms indicated that skilled readers showed somewhat larger responses to words than to pseudowords; while readers with dyslexia did not show such a difference. In addition, a group x pair type interaction (z-score = 3.46, *P*_perm_ = 0.003) between 219 and 273 ms showed that the dyslexia group exhibited greater responses to ID targets compared to SL and PP targets, while the control group did not show a pair type effect. No other effects were significant (lexicality: z-score = 0.77, *P*_perm_ = 0.400; pair type: z-score = 0.84, *P*_perm_ = 0.249; lexicality x pair type: z-score = 0.93, *P*_perm_ = 0.649; group x lexicality x pair type: z-score = 0.65, *P*_perm_ = 0.642).

**Figure 6:**
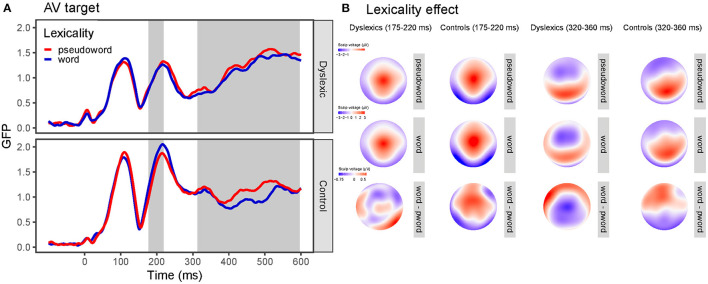
Global field power (GFP) curves and topographic maps for the visual and audiovisual target stimuli. **(A)** GFP for the word and pseudoword targets separately for the dyslexia and control group. Gray areas highlight the time windows in which the effect was significant according to the GFP (first area) and the GMD (second area) analysis. **(B)** Topographic maps for the word and pseudoword targets and their difference (lexicality effect) separately for the dyslexia and control group for the time window where GFP (175–220 ms) and GMD (320–360 ms) differed between the groups.

The GMD analysis revealed a group main effect (z-score = 3.57, *P*_perm_ = 0.003, 284–600 ms), a lexicality main effect (z-score = 4.34, *P*_perm_ = 0.001, 304–600 ms), and a pair type main effect (z-score = 5.47, *P*_perm_ < 0.001, 145–600 ms). Moreover, there was a group x lexicality interaction (z-score = 2.74, *P*_perm_ =0.011, 314–364, 386–437, 463–515, 545–595 ms) and a group x pair type interaction (z-score = 3.87, *P*_perm_ = 0.001, 337–403 ms). The lexicality x pair type (z-score = 0.55, *P*_perm_ = 0.601) and the group x lexicality x pair type interaction (z-score = 0.61, *P*_perm_ = 0.336) were not significant. Topographic maps and GFP curves for the group x lexicality interaction are depicted on Figure 6.

The traditional analysis on the N1 segment on occipital-temporal sites for AV targets (154–292 ms, peak: 216 ms) revealed a main effect of group [*F*_(1, 50)_ = 7.15, *p* =0.010, $\eta_{g}^{2}$ = 0.09] and a four-way interaction of group x lexicality x pair type x laterality [*F*_(2, 100)_ = 3.85, *p* = 0.025, $\eta_{g}^{2}$ = 0.0007]. As simple effect analysis indicated, the interaction was present as there was a pair type x lexicality x laterality interaction for the control group [*F*_(2, 52)_ = 3.56, *p* = 0.046, $\eta_{g}^{2}$ = 0.001] but not for the group with dyslexia [*F*_(2, 48)_ = 0.73, *p* = 0.488, $\eta_{g}^{2}$ = 0.0003]. In the control group, there was a lexicality x pair type interaction [*F*_(2, 52)_ = 3.90, *p* = 0.027, $\eta_{g}^{2}$ = 0.006] due to pair type effect only for words [*F*_(1, 26)_ = 7.80, *p* = 0.010, $\eta_{g}^{2}$ = 0.02] but not for pseudowords [*F*_(1, 26)_ = 0.50, *p* = 0.499, $\eta_{\text{g}}^{2}$ = 0.002] in the left but not in right-hemisphere [*F*_(2, 52)_ = 2.15, *p* = 0.127, $\eta_{g}^{2}$ = 0.004]. The group x lexicality interaction [*F*_(1, 50)_ = 3.12, *p* =0.083, $\eta_{g}^{2}$ = 0.002] just failed to reach significance in this analysis. In addition, all other effects and interactions were non-significant [see (3) Audiovisual processing section in the Supplementary Material].

On the selected frontal-central sites, analysis showed significant main effects of pair type [*F*_(2, 100)_ = 12.67, *p* < 0.001, $\eta_{g}^{2}$ = 0.02] due to differences between ID and IN targets [*p* = 0.017, *p*_*bonferroni*_ = 0.05] but not between IN and PP [*p* =0.41, *p*_*bonferroni*_ = 1.0). The group effect was marginally significant [*F*_(1, 50)_ = 3.82, *p* = 0.056, $\eta_{g}^{2}$ = 0.06]. In addition, there was a group x lexicality interaction [*F*(1, 50) = 6.70, p =0.013, $\eta_{\text{g}}^{2}$ = 0.005) since there was a lexicality effect in the group with dyslexia [*F*_(1, 24)_ = 4.72, *p* = 0.040, $\eta_{g}^{2}$ = 0.006] but not in the control group [*F*_(1, 26)_ = 2.62, *p* = 0.118, $\eta_{g}^{2}$ = 0.004]. The group x pair type interaction just failed to reach significance [*F*_(2, 100)_ = 2.91, *p* =0.059, $\eta_{g}^{2}$ = 0.004]. Furthermore, no other effects were significant [see (3) Audiovisual processing section in the Supplementary Material].

In sum, readers both with and without dyslexia process orthographic stimuli similarly in the visual modality; however, group differences emerged in the AV condition. In the TANOVA analysis, we observed main effects of group and lexicality but no interaction between the two for visual targets. On the other hand, in the audiovisual condition, controls showed a lexicality effect with greater responses for words than for pseudowords, but readers with dyslexia did not. Using the traditional analysis method we found a pair type effect for words in the left-hemisphere for audiovisual targets, but only in readers without dyslexia. However, this effect was not present for visual targets.

## Discussion

In our ERP study, we investigated visual and audiovisual processing of orthographic stimuli by adult readers with and without dyslexia in an implicit same-different task. Our results suggest that (1) readers with and without dyslexia exhibit similar responses to words and pseudowords, (2) readers with and without dyslexia process letter identity and letter position similarly in the visual modality despite topographic differences between the groups, and (3) readers with and without dyslexia exhibit different responses when orthographic stimuli are presented audiovisually. The above results indicate that in developmental dyslexia, orthographic-phonological processing deficits are more pronounced than orthographic processing deficits *per se*.

### Adult With and Without Dyslexia Did Not Show Lexicality Effect

According to our results, orthographic processing is not inefficient in developmental dyslexia. We investigated word and pseudoword processing and expected to find an N1 lexicality effect modulated by reading skill. However, this was not the case as none of the groups showed a lexicality effect. This result is in contrast with some previous studies which reported lexicality effect on N1 in skilled readers but not in readers with dyslexia (Mahé et al., 2012, 2013) but rather supported studies that failed to find differences between the processing of word and pseudoword stimuli (Maurer et al., 2005b, 2006, 2008; Kast et al., 2010; Araújo et al., 2012; Hasko et al., 2013; Eberhard-Moscicka et al., 2015). According to Maurer and McCandliss (2007) lexicality effect occurs when grapheme-phoneme mapping is not automatic, such as in deep orthographies, in novice readers or when an implicit reading tasks is employed. In implicit reading tasks, only those readers can apply grapheme-phoneme mappings whose grapheme-phoneme mappings are fully automatized; therefore, we expected to find lexicality effect only in the group with dyslexia but not in the control group as their grapheme-phoneme mapping should be highly automatized. Therefore, the lack of lexicality effect for skilled readers in our study could be explained by their highly automatic grapheme-phoneme mapping which allows them to read simple pseudowords as efficiently as real words. However, based on the above argument, we expected to find differential processing for words and pseudowords in the dyslexia group whose reading difficulties are characterized by sluggish grapheme-phoneme mapping. Contrary to our hypothesis, the group of readers with dyslexia did not show the lexicality effect either. Visual inspection of the GFP curves (Figure 3) suggests somewhat larger responses for words compared to pseudowords in the N1 time window; however, this difference failed to reach significance in the analysis. Our participants were speakers of a highly transparent language (Hungarian); thus, this could suggest that participants with dyslexia in our study did not have fully automatized grapheme-phoneme mapping, but their decoding is automatic enough due to the shallow orthography of Hungarian so that no lexicality effect could be detected. Our experiment used short, simple pseudowords; however, it is possible that inclusion of longer and more complex pseudowords would result in a stronger lexicality effect especially for the group with dyslexia.

More interestingly, the analysis revealed no group difference in the N1 time window. This is somewhat surprising as most studies conducted with participants with dyslexia reported less left-lateralized effect (Helenius et al., 1999; Kast et al., 2010; Dujardin et al., 2011). However, when the effect was analyzed by applying traditional ERP analysis methods, a significant group x laterality effect emerged in the N1 time window. This indicates that the participants with dyslexia in our study do not have a fully left-lateralized N1 response; however, the difference between the groups is not substantial enough to be detected by the data-driven TANOVA. Although we could not detect differences in orthographic processing it is possible that those group differences are subtle enough so that we cannot capture them in an implicit task.

Taken together, readers with dyslexia process words and pseudowords similarly as typical readers albeit their N1 responses might be less left-lateralized indicating less automatic orthographic-visual mapping but relatively intact orthographic processing.

### Letter Position and Letter Identity Encoding Is Similar in Readers With and Without Dyslexia

To provide further evidence whether inefficient orthographic processing is the main culprit of reading deficits in dyslexia, we compared letter identity and letter order processing of readers with and without dyslexia. In the implicit same-different paradigm, participants were shown stimulus pairs that could be either identical (ID), different in one letter (letter identity neighbor, IN), or different in the position of the letters (letter position pairs, PP). In contrast to our hypothesis that adults with dyslexia show less efficient processing of letter identity and letter order, our results suggest that orthographic processing is similar between the groups.

As opposed to the expected interaction between group and pair type, we found only a main effect of pair type and a main effect of group, but no interaction between them. We are not aware of any previous study that investigated letter identity and letter position coding in adults with dyslexia using EEG, although, previous behavioral studies suggested deficits in letter identity and position processing in dyslexia (Reilhac et al., 2012; Ogawa et al., 2016). However, we did not find any difference in letter identity and letter order processing when word and pseudoword pairs were presented in the visual modality. Furthermore, our results showed that the pair type effect was modulated neither by the lexicality of the stimulus nor by the reading skill of the participants. This result is in contrast with the study of Reilhac et al. (2012) which found a substitution advantage over transposition in controls for both words and pseudowords but in children with dyslexia, the effect was only present for words. Probably, the differences found by Reilhac et al. (2012) arose from the explicit nature of their same-different task. In our paradigm, participants performed a simple feature-detection task which did not require detection of repetition. Thus, the task is less affected by short-term memory load, attentional demands, and strategic top-down effects. It is possible that group differences would emerge when the task requires to hold items in memory or to make strategic decisions. In line with this idea, group differences were found in letter position encoding in a task which used an explicit (naming) task (Ogawa et al., 2016). In addition, when a masked priming paradigm is used (Lété and Fayol, 2013), children with dyslexia show similar performance as same-age peers. This suggests that differences between readers with and without dyslexia in letter identity and letter order processing are not necessarily due to impaired visual word processing, but could be due to attentional factors.

Indeed, visual attentional difficulties have been debated as one of the characteristics in some groups with dyslexia (Valdois et al., 2004). Difficulties in distributing attention during parallel processing could affect letter identification within strings (Reilhac et al., 2012) or ordering of letters leading to sequencing errors (Valdois et al., 2004). Moreover, attentional difficulties may influence parallel processing speed, thus putting more effort in a supposedly automatized task and affecting reading performance (Valdois et al., 2004). Therefore, future studies could explore how much attentional differences contribute to letter identity and position encoding explicitly comparing implicit and explicit reading tasks.

Our results present that groups with and without dyslexia show a similar pair type effect in two time windows. Between 140 and 230 ms identical pairs evoked smaller responses compared to letter identity neighbor or letter position pair targets. In addition, identical targets exhibited different topography. This is in line with previous studies reporting different N1 for identical word pairs compared to different word pairs (Holcomb and Grainger, 2006; Cao et al., 2015). Although Holcomb and Grainger (2006) found that only coarse differences (such as identical vs. different in all letters) modulated N1 responses, they used a time window of 125–175 ms. In a later time window (175–300 ms), they also detected finer word-form differences (identical vs. substituted letter pairs). We applied a data-driven approach of analysis which requires no preselection of specific channels or time windows. The result of TANOVA indicated a time window that partly overlaps with the time windows used by Holcomb and Grainger (2006) which can explain the discrepancies. In addition, Duñabeitia et al. (2012) found greater N1 for substituted letter targets compared to transposed letter targets. Our study replicates these results as letter identity neighbors evoked somewhat smaller responses than letter position pairs. In the later time window (270–600 ms) the identical, letter identity, and letter position pairs showed topographic differences; letter identity neighbors evoked smaller responses than identical or letter position pairs. This is consistent with previous results reporting differential processing of identical and one-letter different prime-target pairs (Holcomb and Grainger, 2006). Duñabeitia et al. (2012) demonstrated larger responses for substituted letter strings than transposed letter strings in a same-different task. It should be noted, however, that they compared two-letter different and transposed letter word pairs, whereas we used one-letter different substitution neighbors. In addition, they found the difference between 200 and 325 ms; whereas the pair type effect in our study was observed in a later time window corresponding to the P3 component.

Compared to the reference stimuli, analysis of the visual targets showed lexicality effect due to greater responses for words compared to pseudowords. Many studies reported greater N1 for pseudowords compared to words (Hauk and Pulvermüller, 2004; Hauk et al., 2006; Dujardin et al., 2011; Araújo et al., 2015); however, the lexicality effect in our study occurred in a later time window (300–400 ms) and is probably related to semantic processing of the real words.

In addition, we found a group main effect. Although control readers showed larger and more left-lateralized responses compared to readers with dyslexia, this difference occurred regardless of stimulus lexicality or pair type. Topographic differences are in line with previous results reporting bilateral N1 response for readers with dyslexia but left-lateralized response for typical readers (Helenius et al., 1999; Kast et al., 2010). As the phonological mapping hypothesis (Maurer and McCandliss, 2007) suggests, left lateralization is driven by automatized grapheme-phoneme mapping which explains why readers who struggle with fluent reading exhibit bilateral responses. Strikingly, the group effect was weak in the reference stimuli; however, target processing enhanced the difference. This could indicate that group differences partly originate from automatic matching of the reference and the target. Namely, the difference extends to later time windows such as the P3 time window which is known to reflect attentional processing related to subsequent memory and stimulus discrimination (Polich, 2007). The divergence, therefore, could signal a general attentional, memory-related difference between the groups.

To summarize, group and lexicality effects were enhanced in the visual targets suggesting that automatic matching of the reference and target stimuli can modulate these effects. In addition, a robust pair type effect was present for adults both with and without dyslexia which signifies that orthographic processing deficit *per se* does not characterize reading deficits in developmental dyslexia.

### Audiovisual Processing Deficits Are More Pronounced Than Orthographic Processing Deficits in Dyslexia

Finally, to investigate whether inefficient orthographic-phonological processing characterizes developmental dyslexia, we presented target stimuli in an audiovisual condition. We assumed that if audiovisual processing is deficient in dyslexia, group differences will be greater in the audiovisual condition compared to the visual only condition. Indeed, our results confirmed this hypothesis. While in the visual condition the dyslexia and control participants showed similar visual word processing as signified by the lack of group x lexicality and group x pair type interaction, in the audiovisual condition group differences emerged.

In the audiovisual condition reading skills modulated the effect of lexicality. Between 178 and 218 ms skilled readers showed larger N1 responses to words than to pseudowords; while readers with dyslexia did not show such a difference. In addition, readers with and without dyslexia showed differential topographic distribution for words and pseudowords after 300 ms due more localized anterior distribution lexicality effect in participants with dyslexia. Previous studies provided mixed results on the emergence and direction of the lexicality effect (no effect: Maurer et al., 2005b, 2006; Araújo et al., 2012; Hasko et al., 2013; Eberhard-Moscicka et al., 2015; greater N1 for words: Maurer et al., 2006; Mahé et al., 2012; Eberhard-Moscicka et al., 2016; greater N1 for pseudowords: Hauk et al., 2006; Dujardin et al., 2011; Araújo et al., 2015) and several factors were proposed which could account for the discrepancies (developmental effects: Eberhard-Moscicka et al., 2016; orthographic depth: Maurer and McCandliss, 2007; and task demands: Faísca et al., 2019). Our study adds to the above results providing evidence that the N1 lexicality effect is not detectable when orthographic stimuli are presented visually in a highly transparent language in an implicit task regardless of reading skills. However, the effect is present when words are presented audiovisually but only for skilled readers. Thus, greater responses to words in our control sample can indicate that audiovisual presentation of words engages reading-related processes automatically. These findings are in accordance with the results of Varga et al. (2020) who reported enhanced N1 effect for audiovisual presentation compared to visual only presentation in typically developing children.

Furthermore, there were group differences in the pair type effect, too. Between 219 and 273 ms, readers with dyslexia exhibited larger responses to identical targets compared to different (letter identity neighbor or letter position pairs), while the control group did not show a pair type effect. In addition, readers with and without dyslexia showed differential topographic distribution from 330 ms. It seems that when the task requires audiovisual processing, the mismatch between the reference and the target stimuli is enhanced for adults with dyslexia. This result seems counterintuitive at first; however, it should be noted that in our paradigm, the auditory stimuli were always identical to the visually presented target stimuli. Therefore, the pair type effect reflected a mismatch between the visual reference and the audiovisual target. Skilled readers showed a robust pair type effect in the visual condition; however, this effect disappeared when the target was presented audiovisually. One possible explanation is that the simultaneous presentation of the same linguistic stimulus in both the visual and audiovisual modality resulted in a prompt integration between the visual target and the auditory target (which were the same) which overrode the integration between reference and target stimuli (which differed). On the other hand, readers with dyslexia showed a pair type effect in the visual condition which was enhanced by the audiovisual presentation. That is, for them probably no automatic integration between the visual target and the auditory target occurred, but the integration between reference and target stimuli was augmented. This hypothesis is supported by previous studies reporting that readers with dyslexia fail to show automatic audiovisual integration when the visual letters and the speech sound are presented simultaneously; however, a weak and late effect of mismatch appeared when the letter appeared 200 ms before the speech sound (Froyen et al., 2011).

Finally, this study provides further support for the findings of Hasko et al. (2012). The researchers tested whether German children with dyslexia demonstrate more severe deficits in a task requiring orthographic-phonological integration than in a task requiring only orthographic processing. They found no group difference in the visual-visual matching task, but children with dyslexia showed different N300 responses when auditory-visual matching was required for word pairs. Our study extends the above finding investigating adult readers with dyslexia in an implicit reading task. Different pair type effect for participants with and without dyslexia could suggest different auditory-visual matching even though this matching was not required by the task. Our results seemingly posit that audiovisual processing deficits can be detected even when the task does not explicitly require grapheme-phoneme binding. Moreover, the results of Hasko et al. (2012) could generalize to pseudoword stimuli since we found the same pattern of results for both word and pseudoword pairs. In addition, not only auditory-visual matching (as in Hasko et al., 2012) but (implicit) visual-auditory matching (as in the present study) is inefficient in developmental dyslexia. Though Hasko et al. (2012) used an explicit auditory-visual matching task where they compared an auditory reference and a visual target whereas our implicit same-different task compared a visual reference and an auditory/visual target, both studies found group differences. Direction of stimulus matching could serve as a future direction of investigation, since the visual-auditory direction is more relevant to reading as graphemes are mapped to phonemes, while the auditory-visual direction is more relevant to spelling as phonemes are mapped to graphemes. Thus, further research could directly compare whether the visual-auditory direction (as in reading) or the auditory-visual direction (as in spelling) is more impaired in dyslexia.

### Limitations

Some limitations of the present study should be noted. First, we did not obtain any measures of IQ; thus, we cannot exclude that group differences result from differences in IQ. However, we believe this is not the case as the groups were recruited with similar level of education and it is not clear how IQ would influence implicit reading skills. Nevertheless, group differences in our study warrant caution as IQ was not controlled.

Second, our study includes five left-handed participants with dyslexia. Although most people have left-hemispheric dominance for language and print processing, left-handed individuals show atypical lateralization more often than right-handed individuals (8 vs. 15%, respectively (Szaflarski et al., 2012). As the incidence of left-handedness in developmental dyslexia is slightly higher than in typical readers (Vlachos et al., 2013), relationship between lateralization and reading difficulties arises. Our results suggested that readers with dyslexia exhibit less left-lateralized N1 responses; however, this could be due to the inclusion of left-handed individuals. Therefore, any lateralization differences between the groups should be interpreted with caution as handedness may confound results.

Third, group differences that result from the comparison of visual and audiovisual processing could be driven by either differences in phonological processing or differences in phonological processing in audiovisual integration. Our experiment does not allow differentiating between the two alternative explanations. Though impaired phonological processing and impaired orthographic-phonological mapping seem to be related (Blomert, 2011), future studies should follow up by comparing the audiovisual condition to both visual and auditory unimodal conditions to further investigate audiovisual integration in developmental dyslexia.

### Conclusion

In conclusion, in an implicit same-different task we could not demonstrate any orthographic processing deficit such as fine-grained print sensitivity or letter identity and letter position encoding deficit in adult readers with dyslexia. However, we found group differences in audiovisual stimulus processing suggesting that in dyslexia phonological and orthographic-phonological processing deficits are more fundamental than orthographic-visual deficits.

## Data Availability Statement

The dataset used in this study is available at: https://osf.io/6bt8g/?view_only=b1c6684655da4e9194b1e36b0f7430b9.

## Ethics Statement

The studies involving human participants were reviewed and approved by United Ethical Review Committee for Research in Psychology. The patients/participants provided their written informed consent to participate in this study.

## Author Contributions

DT and VC: conceptualization. DT: experimental task preparation. DC: data collection. VV, KA, DC, and DT: analysis. VV: writing—original draft. VV, KA, DT, DC, and VC: writing—revision and editing. All authors contributed to the article and approved the submitted version.

## Funding

This research was funded by the Hungarian Science Foundation (OTKA NK 101087 and K 119365) and the Neo-PRISM-C project (European Union Horizon 2020 Program, H2020-MSCA-ITN-2018) under the Marie Skłodowska-Curie Innovative Training Network (Grant Agreement No. 813546).

## Conflict of Interest

The authors declare that the research was conducted in the absence of any commercial or financial relationships that could be construed as a potential conflict of interest.

## Publisher's Note

All claims expressed in this article are solely those of the authors and do not necessarily represent those of their affiliated organizations, or those of the publisher, the editors and the reviewers. Any product that may be evaluated in this article, or claim that may be made by its manufacturer, is not guaranteed or endorsed by the publisher.
