# Supplementary material for: ERP Correlates of Altered Orthographic-Phonological Processing in Dyslexia
Source: Front Psychol. 2021 Oct 13;12:723404. doi: 10.3389/fpsyg.2021.723404 (PMC8548581; doi:10.3389/fpsyg.2021.723404)
Supplement: Supplementary file 1 [file Data_Sheet_1.docx]

Supplementary Material

Table S1. Stimuli of the experiment. Base: Base word that served as the reference stimulus; Targets could be ID: identical; IN: letter identity neighbor; or PP: letter position pair. Filler items are presented in bold fonts.

| **Word** | | | | **Pseudoword** | | | |
| --- | --- | --- | --- | --- | --- | --- | --- |
| **Base** | **ID** | **IN** | **PP** | **Base** | **ID** | **IN** | **PP** |
| BÁL | BÁL | LÁB | SÁL | DÁL | DÁL | LÁD | ZÁL |
| CÉL | CÉL | LÉC | DÉL | CÉZ | CÉZ | ZÉC | DÉZ |
| ÉLE | ÉLE | ELÉ | ÖLE | ÁNA | ÁNA | ANÁ | UNA |
| GÓL | GÓL | LÓG | JÓL | GÓN | GÓN | NÓG | KÓN |
| HAS | HAS | SAH | SAS | HES | HES | SEH | SES |
| KAR | KAR | RAK | MAR | SAR | SAR | RAS | DAR |
| KÉP | KÉP | PÉK | LÉP | RÉP | RÉP | PÉR | SÉP |
| LÁT | LÁT | TÁL | FÁT | KÁJ | KÁJ | JÁK | SÁJ |
| LÉG | LÉG | GÉL | VÉG | KÉG | KÉG | GÉK | SÉG |
| LÚD | LÚD | DÚL | RÚD | LOD | LOD | DOL | SOD |
| MÁR | MÁR | RÁM | SÁR | NÁR | NÁR | RÁN | FÁR |
| MÉG | MÉG | GÉM | JÉG | TÉG | TÉG | GÉT | HÉG |
| MÉR | MÉR | RÉM | FÉR | MÖR | MÖR | RÖM | PÖR |
| MÓD | MÓD | DÓM | HÓD | NÓD | NÓD | DÓN | PÓD |
| MŰT | MŰT | TŰM | FŰT | GŰT | GŰT | TŰG | ZŰT |
| NÁD | NÁD | DÁN | VÁD | PÁD | PÁD | DÁP | JÁD |
| NÉV | NÉV | VÉN | HÉV | MÉV | MÉV | VÉM | DÉV |
| RÁK | RÁK | KÁR | MÁK | GÁK | GÁK | KÁG | VÁK |
| RÁZ | RÁZ | ZÁR | HÁZ | RÓZ | RÓZ | ZÓR | HÓZ |
| SÁS | SÁS | ÁSS | MÁS | SÚS | SÚS | ÚSS | MÚS |
| SOK | SOK | KOS | FOK | SOL | SOL | LOS | FOL |
| SOM | SOM | MOS | ROM | SOT | SOT | TOS | MOT |
| TÁG | TÁG | GÁT | RÁG | BÁG | BÁG | GÁB | MÁG |
| TÁR | TÁR | ÁRT | VÁR | TÓR | TÓR | ÓRT | VÓR |
| TEJ | TEJ | EJT | FEJ | TER | TER | ERT | GER |
| TÉL | TÉL | ÉLT | BÉL | TEL | TEL | ELT | DEL |
| TÉR | TÉR | RÉT | KÉR | TÉS | TÉS | SÉT | FÉS |
| TÍZ | TÍZ | ÍZT | VÍZ | TÚZ | TÚZ | ÚZT | RÚZ |
| TOL | TOL | OLT | HOL | TOJ | TOJ | OJT | ZOJ |
| TÖK | TÖK | KÖT | LÖK | FÖK | FÖK | KÖF | RÖK |
| TŐR | TŐR | RŐT | BŐR | GŐR | GŐR | RŐG | VŐR |
| TÚR | TÚR | RÚT | FÚR | TÚM | TÚM | MÚT | RÚM |
| TŰR | TŰR | ŰRT | ZŰR | TÖJ | TÖJ | ÖJT | ZÖJ |
| ÜST | ÜST | SÜT | EST | ÖST | ÖST | SÖT | AST |
| VAS | VAS | SAV | KAS | VAZ | VAZ | ZAV | NAZ |
| VÉR | VÉR | ÉRV | BÉR | GÉR | GÉR | ÉRG | NÉR |
| **AMI** | **AMI** | **IMA** | **AKI** | **ASI** | **ASI** | **ISA** | **ÜSI** |
| **BÖK** | **BÖK** | **KÖB** | **BÓK** | **BÖT** | **BÖT** | **TÖB** | **LÖT** |
| **ESŐ** | **ESŐ** | **ŐSE** | **ERŐ** | **ASÓ** | **ASÓ** | **ÓSA** | **USÓ** |
| **ÓDA** | **ÓDA** | **ADÓ** | **ODA** | **ÓFA** | **ÓFA** | **AFÓ** | **OFA** |
| ÁRAM | ÁRAM | MÁRA | IRAM | ARUM | ARUM | MURA | ORUM |
| ÁRAT | ÁRAT | ARÁT | ARAT | ÓROT | ÓROT | ORÓT | OROT |
| ÁRVA | ÁRVA | VÁRA | ÍRVA | ÁRNA | ÁRNA | NÁRA | ORNA |
| BABA | BABA | ABBA | BÁBA | ZAZA | ZAZA | AZZA | KAZA |
| BETŰ | BETŰ | TŰBE | TETŰ | BEVŰ | BEVŰ | VŰBE | VEVŰ |
| BIKA | BIKA | BAKI | BOKA | DIKA | DIKA | DAKI | DOKA |
| BUTA | BUTA | TABU | GUTA | DUTA | DUTA | TADU | ZUTA |
| DARU | DARU | URAD | SARU | BARU | BARU | URAB | NARU |
| EGÉR | EGÉR | ÉGRE | ÍGÉR | EGŐR | EGŐR | ŐGRE | ÜGŐR |
| ELEM | ELEM | EMEL | EREM | ULUM | ULUM | UMUL | URUM |
| ÉLET | ÉLET | ÉTEL | ÉGET | ŰLET | ŰLET | ŰTEL | ŰLES |
| ERŐD | ERŐD | ERDŐ | ERED | ENŐD | ENŐD | ENDŐ | ENED |
| ESTE | ESTE | ESET | ESNE | ASTA | ASTA | ASAT | ASNA |
| IRKA | IRKA | IKRA | INKA | IRDA | IRDA | IDRA | IRBA |
| IZOM | IZOM | MOZI | ÁZOM | IZON | IZON | NOZI | UZON |
| KAJA | KAJA | AJAK | MAJA | KAGA | KAGA | AGAK | FAGA |
| KARÓ | KARÓ | RÓKA | MARÓ | TARÓ | TARÓ | RÓTA | NARÓ |
| KÉNE | KÉNE | ÉNEK | NÉNE | KÁNA | KÁNA | ÁNAK | RÁNA |
| KENI | KENI | NEKI | FENI | TENI | TENI | NETI | VENI |
| KÉRI | KÉRI | ÉRIK | TÉRI | PÉRI | PÉRI | ÉRIP | FÉRI |
| KÓLA | KÓLA | LAKÓ | RÓLA | SÓLA | SÓLA | LASÓ | MÓLA |
| KUPA | KUPA | KAPU | KAPA | TUPA | TUPA | TAPU | TAPA |
| LIGA | LIGA | ALIG | GIGA | LEGA | LEGA | ALEG | LELA |
| MÓKA | MÓKA | KÓMA | HÓKA | NÓKA | NÓKA | KÓNA | SÓKA |
| ÖRÖK | ÖRÖK | ÖKÖR | ÖNÖK | ÜRÜK | ÜRÜK | ÜKÜR | ÜNÜK |
| PALA | PALA | ALAP | HALA | JALA | JALA | ALAJ | GALA |
| PÁRA | PÁRA | ÁRPA | KÁRA | DÁRA | DÁRA | ÁRDA | DÁVA |
| PIPA | PIPA | PAPI | PÁPA | BIBA | BIBA | BABI | BÓBA |
| SUTA | SUTA | SATU | SÉTA | SÓTA | SÓTA | SATÓ | SITA |
| TAVI | TAVI | VITA | HAVI | TEVI | TEVI | VITE | HEVI |
| TELT | TELT | LETT | KELT | TALT | TALT | LATT | KALT |
| VALÓ | VALÓ | ALVÓ | FALÓ | GALÓ | GALÓ | ALGÓ | ZALÓ |
| VÁZA | VÁZA | ÁZVA | HÁZA | VÉZE | VÉZE | ÉZVE | HÉZE |
| VELE | VELE | LEVE | TELE | ZELE | ZELE | LEZE | SELE |
| VELŐ | VELŐ | LEVŐ | LELŐ | RELŐ | RELŐ | LERŐ | NELŐ |
| VERT | VERT | TERV | KERT | VORT | VORT | TORV | NORT |
| **AGÁR** | **AGÁR** | **ÁGRA** | **AKÁR** | **AJÁR** | **AJÁR** | **ÁJRA** | **OJÁR** |
| **DIÓS** | **DIÓS** | **ÓSDI** | **DIÓT** | **PIÓS** | **PIÓS** | **ÓSPI** | **PIÓT** |
| **FAKÓ** | **FAKÓ** | **FÓKA** | **ZAKÓ** | **FEKÓ** | **FEKÓ** | **FÓKE** | **ZEKÓ** |
| **RÉTI** | **RÉTI** | **ÉRTI** | **RÉGI** | **NÉTI** | **NÉTI** | **ÉNTI** | **NÉGI** |
| ÁLLAT | ÁLLAT | ÁLTAL | ÁLTAT | ÓLLAT | ÓLLAT | ÓLTAL | ÓLTAT |
| BÁTOR | BÁTOR | TÁBOR | SÁTOR | DÁTOR | DÁTOR | TÁDOR | ZÁTOR |
| DÖRÖG | DÖRÖG | ÖRDÖG | PÖRÖG | BÖRÖG | BÖRÖG | ÖRBÖG | VÖRÖG |
| GÖRBE | GÖRBE | BÖGRE | KÖRBE | GÖRPE | GÖRPE | PÖGRE | HÖRPE |
| HATÁS | HATÁS | HÁTAS | PATÁS | HETÉS | HETÉS | HÉTES | RETÉS |
| JELÉN | JELÉN | ÉLJEN | FELÉN | JALÁN | JALÁN | ÁLJAN | VALÁN |
| KÉREK | KÉREK | KERÉK | KEREK | KÁNAK | KÁNAK | KANÁK | KANAK |
| KEZEM | KEZEM | MEZEK | MEZEM | KEFEM | KEFEM | MEFEK | MEFEM |
| KIÁLT | KIÁLT | KILÁT | KIOLT | KIÁJT | KIÁJT | KIJÁT | KIOJT |
| LAKÁS | LAKÁS | SAKÁL | RAKÁS | LEKÉS | LEKÉS | SEKÉL | REKÉS |
| LÁMPA | LÁMPA | PÁLMA | RÁMPA | LÁMBA | LÁMBA | BÁLMA | ZÁMBA |
| LAPKA | LAPKA | KALAP | SAPKA | RAPKA | RAPKA | KARAP | DAPKA |
| LÉLEK | LÉLEK | LÉKEL | LÉPEK | LÁLAK | LÁLAK | LÁKAL | LÁMAK |
| MÁLNA | MÁLNA | NÁLAM | BÁLNA | MÁGNA | MÁGNA | NÁGAM | BÁGNA |
| MANGÓ | MANGÓ | MAGNÓ | MARGÓ | MINGÓ | MINGÓ | MIGNÓ | MIRGÓ |
| MÁRKA | MÁRKA | KARÁM | BÁRKA | NÁRKA | NÁRKA | KARÁN | FÁRKA |
| MAROK | MAROK | KAROM | FAROK | MAROZ | MAROZ | ZAROM | GAROZ |
| MEREK | MEREK | REMEK | BEREK | MARAK | MARAK | RAMAK | VARAK |
| NŐKET | NŐKET | TEKNŐ | FŐKET | SŐLET | SŐLET | TELSŐ | RŐLET |
| OLVAS | OLVAS | LOVAS | ODVAS | OLKAS | OLKAS | KOVAS | ULKAS |
| RAJTA | RAJTA | TARAJ | PAJTA | RANTA | RANTA | TARAN | PANTA |
| REDŐI | REDŐI | IDŐRE | FEDŐI | TEPŐI | TEPŐI | IPŐTE | FEPŐI |
| RÉGEN | RÉGEN | NÉGER | CÉGEN | RÁGAN | RÁGAN | NÁGAR | VÁGAN |
| RÉMES | RÉMES | ÉRMES | FÉMES | RÁMAS | RÁMAS | ÁRMAS | HÁMAS |
| RÉSEK | RÉSEK | ÉRSEK | KÉSEK | RÁSOK | RÁSOK | ÁRSOK | VÁSOK |
| RETEK | RETEK | TEKER | CETEK | PETEK | PETEK | TEKEP | SETEK |
| TÁBLA | TÁBLA | TÁLBA | TÁMLA | KUBLA | KUBLA | KULBA | KUMLA |
| TALÁN | TALÁN | LÁTNA | FALÁN | TAMÁN | TAMÁN | MÁTNA | GAMÁN |
| TAPOS | TAPOS | POSTA | LAPOS | TADOS | TADOS | DOSTA | LADOS |
| TARKA | TARKA | TAKAR | BARKA | TANKA | TANKA | TAKAN | FANKA |
| TEHET | TEHET | HETET | MEHET | TAHAT | TAHAT | HATAT | MAHAT |
| TELEK | TELEK | KELET | JELEK | TELEZ | TELEZ | ZELET | KELEZ |
| TÉLEN | TÉLEN | ÉTLEN | DÉLEN | KÉLEN | KÉLEN | ÉKLEN | PÉLEN |
| TEREK | TEREK | KERET | PEREK | TEMEK | TEMEK | KEMET | JEMEK |
| VÉSEK | VÉSEK | KEVÉS | RÉSEK | MÉSEK | MÉSEK | KEMÉS | PÉSEK |
| VONAT | VONAT | TAVON | FONAT | ZONAT | ZONAT | TAZON | BONAT |
| **BOROK** | **BOROK** | **BOKOR** | **BUROK** | **LOGOR** | **LOGOR** | **LOROG** | **LOHOR** |
| **KAPUS** | **KAPUS** | **PUSKA** | **KAPÁS** | **KAGUS** | **KAGUS** | **GUSKA** | **KAGÁS** |
| **KETTŐ** | **KETTŐ** | **TETŐK** | **KETTÉ** | **KÜTTŐ** | **KÜTTŐ** | **KÜTŐK** | **KÜTTÉ** |
| **TÁJÉK** | **TÁJÉK** | **JÁTÉK** | **TÁJAK** | **VÁJÉK** | **VÁJÉK** | **JÁVÉK** | **SÁJÉK** |

*(1) Lexicality effect*

The GFP segmentation resulted in lengthy N1 segment for the reference stimuli (139 – 324 ms, peak: 223 ms). Due to the long latency of the GFP windows, we divided the N1 time window into two parts to check the specific stages the effect occur, which indicates an early N1 (139 - 223 ms), primarily responsible for coarse-print tuning, and a late N1 (223- 324 ms), believed to be more sensitive to fine tuning (e.g., lexicality effect, see Eberhard-Moscicka, Jost, Fehlbaum, Pfenninger, and Maurer, 2016). For N1 lexicality analysis, we formed channel clusters from occipito-temporal channels (left (LH): O1, P7, right (RH): O2, P8, based on (Kast, Elmer, Jancke, & Meyer, 2010).

The traditional analysis in the reference stimuli (139 – 324 ms) showed similar results in both the early and late N1 time windows. In the early N1 (139 - 223 ms) time window, only laterality (*F* (1, 50) = 8.30, p= .006, η^2^_g_ = 0.02) and group x laterality (*F*(1, 50) = 4.76, p = .034, η^2^_g_  = 0.001) were significant. Effect of group (*F*(1, 50) = 0.16, p =.687, η^2^_g_ = 0.003), lexicality (*F*(1, 50) = 0.73, p = .398, η^2^_g_  = 0.0006), group x lexicality (*F*(1, 50) = 3.01, p =.089, η^2^_g_  = 0.003), lexicality x laterality (*F*(1,50) = 0.97, p =.331, η^2^_g_  = 0.0001), or group x lexicality x laterality (*F*(1, 50) = 0.01, p =.936, η^2^_g_ = 0.000001) were not significant.

Similarly, in the late N1 (223- 324 ms) time window, the main effect of laterality (*F* (1, 50) =7.46, p = .009, η^2^_g_ = 0.02) and group x laterality (*F*(1, 50) = 4.56, p = .038, η^2^_g_ = 0.01) interaction was significant. In addition, there was also a significant main effect of group (*F*(1, 50) = 4.88, p = .032, η^2^_g_  = 0.07). Once again, lexicality (*F*(1, 50) = 1.03, p = .314, η^2^_g_ = 0.001), group x lexicality (*F*(1, 50) = 3.14, p = .083, η^2^_g_ = 0.003), lexicality x laterality (*F*(1, 50) = 1.37, p = .247, η^2^_g_  = 0.0001), and group x lexicality x laterality interaction (*F*(1,50) = 0.49, p =.485, η^2^_g_ = 0.0004) did not reach significance.

*(2) Letter identity and position encoding*

The traditional analysis on the N1 segment on occipital-temporal sites for visual targets (140-304 ms, peak: 231 ms) revealed a significant effect on group (*F*(1, 50) = 6.81, p = .012 , η^2^_g_ = 0.08), wherein controls showed more negative responses than dyslexics. Moreover, the interaction between lexicality and laterality (*F*(1, 50) = 5.23, p = .027 , η^2^_g_ = 0.001) was also significant. The interaction was present as word targets were somewhat more left-lateralized than pseudoword targets, though laterality was not significant for either the words (*F*(1, 50) = 3.34, p = .073 , η^2^_g_ = 0.01) or the pseudowords (F(1, 50) = 0.34, p = .561, η^2^_g_ = 0.0008) when analyzed separately. The main effect of pair type was marginally significant (*F*(2, 100) = 3.04, p = .0525 , η^2^_g_ = 0.004). The pair type effect suggested that ID targets elicited somewhat less negative response than PP (p = .15, p_bonferroni_ = .44) or IN (p = .20, p_bonferroni_ = .61), while PP and IN did not differ from each other (p = 0.85, p_bonferroni_ = 1.0). No other effects or interactions were significant (lexicality: *F*(1, 50) = 0.34, p = .565, η^2^_g_ = 0.0004; laterality: *F*(1, 50) = 1.71, p = .198, η^2^_g_ = 0.004; group x lexicality: *F*(1, 50) = 1.79, p = .187, η^2^_g_ = 0.002; group x pair type: *F*(2, 100) = 1.02, p = .365, η^2^_g_ = 0.001; group x laterality: *F*(1, 50) = 2.44, p = .125, η^2^_g_ = 0.006; lexicality x pair type: *F*(2, 100) = 1.30, p = .277, η^2^_g_ = 0.001; pair type x laterality: F(2, 100) = 2.17, p = .120, η^2^_g_ = 0.0005; group x lexicality x pair type: *F*(2, 100) = 2.71, p = .072, η^2^_g_ = 0.003; group x lexicality x laterality: *F*(1, 50) = 0.90, p = .346, η^2^_g_ = 0.0002; group x pair type x laterality: *F*(2, 100) = 0.70, p = .498, η^2^_g_ = 0.0002; lexicality x pair type x laterality: *F*(2, 100) = 1.16, p = .317, η^2^_g_ = 0.0002; group x lexicality x pair type x laterality: *F*(2, 100) = 0.35, p = .707 , η^2^_g_ = 0.00006).

On the selected frontal-central sites, repeated measures ANOVA showed significant main effects on group (*F*(1, 50) = 8.53 , p =0.005, η^2^_g_ = 0.10), in which controls generated a bigger response than dyslexics. In addition, the pair type effect was marginally significant (*F*(2, 100) = 3.02, p = 0.053, η^2^_g_ = 0.005). Furthermore, neither the lexicality effect (*F*(1, 50) = 0.50, p = .485, η^2^_g_ = 0.001) nor the interactions (group x lexicality: *F*(1, 50) = 0.25, p = .616, η^2^_g_ = 0.0007; group x pair type: *F*(2, 100) = 0.04, p = .958, η^2^_g_ = 0.00006; lexicality x pair type: *F*(2, 100) = 1.34, p = .268, η^2^_g_ = 0.003; group x lexicality x pair type: *F*(2, 100) = 0.75, p = .476, η^2^_g_ = 0.002) showed any significant effect.

(*3) Audiovisual processing*

The traditional analysis on the N1 segment on occipital-temporal sites for AV targets (154-292 ms, peak: 216 ms) revealed a main effect of group (*F*(1, 50) = 7.15, p = .010, η^2^_g_ = 0.09) and a four-way interaction of group x lexicality x pair type x laterality (*F*(2, 100) = 3.85, p = .025, η^2^_g_ = 0.0007). As simple effect analysis indicated, the interaction was present as there was a pair type x lexicality x laterality interaction for the controls (*F*(2, 52) = 3.56, p = .046, η^2^_g_ = 0.001) but not for the dyslexics (*F*(2, 48) = 0.73, p = .488, η^2^_g_ = 0.0003). In the control group, there was a lexicality x pair type interaction (*F*(2, 52) = 3.90, p = .027, η^2^_g_ = 0.006) due to pair type effect only for words (*F*(1, 26) = 7.80, p = .010, η^2^_g_ = 0.02) but not for pseudowords (*F*(1, 26) = 0.50, p = .499, η^2^_g_ = 0.002) in the left but not in right-hemisphere (*F*(2, 52) = 2.15, p = .127, η^2^_g_ = 0.004). The group x lexicality interaction (*F*(1, 50) = 3.12, p = .083, η^2^_g_ = 0.002) just failed to reach significance in this analysis. In addition, all other effects and interactions were non-significant (lexicality: *F*(1, 50) = 1.78, p = .188, η^2^_g_ = 0.001; pair type: *F*(2, 100) = 1.57, p = .214, η^2^_g_ = 0.001; laterality: F(1, 50) = 0.49, p = .486, η^2^_g_ = 0.001; group x pair type: *F*(2, 100) = 0.56, p = .575, η^2^_g_ = 0.0005; group x laterality: *F*(1, 50) = 0.87, p = .355, η^2^_g_ = 0.002; lexicality x pair type: *F*(2, 100) = 1.01, p = .368, η^2^_g_ = 0.001; lexicality x laterality *F*(1, 50) = 0.07, p = .792, η^2^_g_ = 0.00002; pair type x laterality: *F*(2, 100) = 0.76, p = .471, η^2^_g_ = 0.0002; group x lexicality x pair type: *F*(2, 100) = 1.64, p = .199, η^2^_g_ = 0.001; group x lexicality x laterality: *F*(1, 50) = 0.13, p = .724, η^2^_g_ = 0.00003; group x pair type x laterality: *F*(2, 100) = 0.36, p = .701, η^2^_g_ = 0.0001; lexicality x pair type x laterality: *F*(2, 100) = 0.88, p = .418, η^2^_g_ = 0.0002).

On the selected frontal-central sites, analysis showed significant main effects of pair type (*F*(2, 100) = 12.67, p < .001, η^2^_g_ = 0.02) due to differences between ID and IN targets (p = .017, p_bonferroni_ = .05) but not between IN and PP (p = .41, p_bonferroni_ = 1.0). The group effect was marginally significant (*F*(1, 50) = 3.82, p = .056, η^2^_g_ = 0.06). In addition, there was a group x lexicality interaction (*F*(1, 50) = 6.70, p = .013, η^2^_g_ = 0.005) since there was a lexicality effect in the group with dyslexia (*F*(1, 24) = 4.72, p = .040, η^2^_g_ = 0.006) but not in the control group (*F*(1, 26) = 2.62, p = .118, η^2^_g_ = 0.004). The group x pair type interaction just failed to reach significance (*F*(2, 100) = 2.91, p = .059, η^2^_g_ = 0.004). Furthermore, neither the lexicality effect (*F*(1, 50) = 0.001, p = .977, η^2^_g_ = 0.000001) nor the other interactions (lexicality x pair type: *F*(2, 100) = 1.05, p = .354, η^2^_g_ = 0.002; group x lexicality x pair type: *F*(2, 100) = 0.08, p = .924, η^2^_g_ = 0.0001) showed any significant effect.
